# Modeling the Impact of a Highly Potent *Plasmodium falciparum* Transmission-Blocking Monoclonal Antibody in Areas of Seasonal Malaria Transmission

**DOI:** 10.1093/infdis/jiad101

**Published:** 2023-04-12

**Authors:** Joseph D Challenger, Stijn W van Beek, Rob ter Heine, Saskia C van der Boor, Giovanni D Charles, Merel J Smit, Chris Ockenhouse, John J Aponte, Matthew B B McCall, Matthijs M Jore, Thomas S Churcher, Teun Bousema

**Affiliations:** Medical Research Council Centre for Global Infections Disease Analysis, Department of Infectious Disease Epidemiology, Imperial College London, London, United Kingdom; Department of Pharmacy, Radboud Institute for Health Sciences; Department of Pharmacy, Radboud Institute for Health Sciences; Department of Medical Microbiology, Radboud University Medical Center, Nijmegen, The Netherlands; Medical Research Council Centre for Global Infections Disease Analysis, Department of Infectious Disease Epidemiology, Imperial College London, London, United Kingdom; Department of Medical Microbiology, Radboud University Medical Center, Nijmegen, The Netherlands; PATH Center for Vaccine Innovation and Access, Washington, District of Columbia, USA; PATH Center for Vaccine Innovation and Access, Geneva, Switzerland; Department of Medical Microbiology, Radboud University Medical Center, Nijmegen, The Netherlands; Department of Medical Microbiology, Radboud University Medical Center, Nijmegen, The Netherlands; Medical Research Council Centre for Global Infections Disease Analysis, Department of Infectious Disease Epidemiology, Imperial College London, London, United Kingdom; Department of Medical Microbiology, Radboud University Medical Center, Nijmegen, The Netherlands

**Keywords:** infectious reservoir of malaria, malaria modeling, malaria transmission, monoclonal antibodies, pharmacokinetic/pharmacodynamic modeling

## Abstract

Transmission-blocking interventions can play an important role in combating malaria worldwide. Recently, a highly potent *Plasmodium falciparum* transmission-blocking monoclonal antibody (TB31F) was demonstrated to be safe and efficacious in malaria-naive volunteers. Here we predict the potential public health impact of large-scale implementation of TB31F alongside existing interventions. We developed a pharmaco-epidemiological model, tailored to 2 settings of differing transmission intensity with already established insecticide-treated nets and seasonal malaria chemoprevention interventions. Community-wide annual administration (at 80% coverage) of TB31F over a 3-year period was predicted to reduce clinical incidence by 54% (381 cases averted per 1000 people per year) in a high-transmission seasonal setting, and 74% (157 cases averted per 1000 people per year) in a low-transmission seasonal setting. Targeting school-aged children gave the largest reduction in terms of cases averted per dose. An annual administration of the transmission-blocking monoclonal antibody TB31F may be an effective intervention against malaria in seasonal malaria settings.

## Teaser

Monoclonal antibodies could become a valuable tool against malaria. Here we utilized results from the first-in-human study for a transmission-blocking monoclonal antibody to predict its potential public health impact. We also identified age groups to prioritize with this intervention.

Malaria remains a major health problem and caused approximately 627 000 deaths in 2020 [1]. Considerable progress has been made in reducing the malaria burden [2], and the recent recommendation of the World Health Organization to implement vaccination with the first-ever malaria vaccine, RTS,S/AS01, fuels optimism for further successes. However, progress has slowed in recent years [1] and the emergence and spread of insecticide and drug resistance threaten the efficacy of the interventions responsible for much of the recent progress in malaria control [3, 4]. New interventions that reduce the transmission of malaria are high on the priority list of tools for malaria control and eradication [5], as well as in the containment of drug-resistant malaria.

Transmission-blocking vaccines aim to elicit antibodies that interfere with the transmission of malaria to mosquitoes by preventing fertilization of *Plasmodium* transmission stages, gametes, or later sporogonic development in the mosquito gut [6]. The immediate consequence of vaccination is an antibody-mediated reduction in the infection (oocyst) burden in mosquitoes. The reduction in oocyst density defines the transmission-reducing activity (TRA). TRA is closely associated with the reduction in the proportion of mosquitoes that become infected (transmission-blocking activity [TBA]) [7]. By reducing the number of infected mosquitoes, transmission-blocking vaccines reduce malaria incidence at the community level. Transmission-blocking vaccines based on prefertilization gametocyte antigens Pfs230 and Pfs48/45 are currently in clinical trials [6, 8]. These vaccines could be deployed in combination with anti-infection vaccines to increase their community impact [9]. Instead of inducing antibodies by active immunization, monoclonal antibodies (mAbs) targeting the same antigens may be directly administered to achieve the same impact.

TB31F is a humanized version of the highly potent transmission-blocking rat mAb 85RF45.1 [10–13]. It targets a highly conserved epitope on Pfs48/45, expressed on *Plasmodium falciparum* mature gametocytes and gametes. In a recent first-in-human study, an intravenous dose up to 10 mg/kg was well-tolerated in adult study participants with minimal to no side effects [14]. In the highest dose group, serum from trial participants fully prevented transmission to mosquitoes in ex vivo assays throughout 84 days of follow-up. Extrapolation from the estimated TB31F half-life suggests that a single administration could span most of the malaria transmission season in many regions [15], making it attractive from an implementation perspective. It is unclear what dose would be required to obtain effective transmission reduction throughout the malaria season, what the expected community impact would be, and which age groups should be targeted to provide maximum impact. Demographically targeted interventions are operationally attractive but require an understanding of what populations are most important for transmission to mosquitoes [16, 17].

We sought to predict the potential public health impact of different implementation strategies with this transmission-blocking mAb, combining a pharmacological model describing the TB31F exposure–response relationship with a dynamic model of malaria transmission. This pharmaco-epidemiological model allowed us to predict the potential impact of TB31F alongside established public health interventions against malaria, in 2 highly seasonal settings with differing transmission intensity.

## METHODS

### Data

We used data from a first-in-human, dose-escalation study of TB31F that was performed in 25 healthy adult malaria-naive volunteers [14]. There were 5 study arms (n = 5 per arm) with escalating TB31F dose: 4 groups received intravenous TB31F at 0.1 mg/kg, 1 mg/kg, 3 mg/kg, and 10 mg/kg; a fifth group received 100 mg TB31F subcutaneously. Serum for pharmacokinetic and pharmacodynamic analysis was collected before administration; upon the end of infusion; at 1, 3, and 6 hours and on days 1, 2, 7, 14, 21, 28, 56, and 84 after the end of administration. TB31F serum concentrations were quantified by an enzyme-linked immunosorbent assay against the recombinant protein R0.6C that contains the 6C fragment of the Pfs48/45 antigen to which TB31F binds. TRA was determined by standard membrane feeding assay (SMFA) with cultured *P falciparum* gametocytes and laboratory-reared *Anopheles stephensi* mosquitoes [18, 19]. The SMFA measured the TRA in mosquitoes fed on gametocytes in the presence of participants’ serum compared to pooled naive serum. One participant in the subcutaneous group was excluded from all analyses due to implausibly low TB31F concentrations, potentially following incorrect administration.

### Pharmacokinetic/Pharmacodynamic Modeling

Parametric nonlinear mixed-effects modeling was performed using NONMEM version 7.4.1 to analyze the pharmacokinetic data and the relationship with TRA [20] (Supplementary Methods). Weight-based intravenous dosing regimens were explored, aiming to maintain a TRA >80% for a duration similar to that estimated from the trial data for a 70-kg adult administered 10 mg/kg TB31F intravenously.

### Predicting Public Health Impact Using Transmission Modeling

To explore the potential epidemiological impact of TB31F, we incorporated the results of the pharmacokinetic-pharmacodynamic model into a malaria transmission model by Griffin et al [21, 22]. The model allows the introduction of interventions against malaria, such as insecticide-treated nets (ITNs) and seasonal malaria chemoprevention (SMC). This model is described in detail elsewhere [21, 22], and is outlined in the Supplementary Methods.

In previous work [23], a model of a hypothetical transmission-blocking vaccine was added to the transmission model to predict the impact such a vaccine could have and to identify key age groups to vaccinate. Unlike the previous work, the framework used here allows for interindividual variation in the pharmacokinetics, as well as weight-dependent dosing. In the transmission model, the age of individuals is tracked, not their weight; we therefore utilized a weight-for-age model (see Wasmann et al [24] and Supplementary Methods) to facilitate weight-based dosing. Within the transmission model, the TB31F concentration is described by the pharmacokinetic model (Supplementary Table 1).

We investigated the impact of introduction of TB31F in African settings with seasonal malaria transmission, contrasting a low-endemicity (Upper River region in The Gambia) and high-endemicity site (Sahel region in Burkina Faso). In the Supplementary Materials, we describe the seasonality (based on rainfall patterns [25]), transmission intensity (based on *P falciparum* parasite prevalence in 2- to 10-year-olds [PfPR_2-10_] [26]), and coverage and efficacy of ITNs, antimalarial treatment [26], and SMC [1]. We then varied the mosquito-to-human ratio to obtain the desired level of endemicity. In the Sahel region of Burkina Faso, the mean PfPR_2-10_ in 2019 was 20%, and as high as 30% in some locations. We elected to model a site with a relatively high prevalence for the region: We set a baseline entomological inoculation rate (EIR) of 60 infectious bites per person per year (ibpppy), resulting in a modeled PfPR_2-10_ of 29%. In the Upper River region of the Gambia, the observed PfPR_2-10_ was much lower (average value across the region in 2019 was 6%, which we matched using a baseline EIR of 7 ibpppy).

TB31F was administered annually, concurrent with the first SMC dose: this time point was chosen to optimize SMC efficacy for the seasonality of each region. In our default scenario, against which the impact of SMC and TB31F are measured, SMC is withdrawn for a 3-year period, leaving ITNs as the only public health intervention (other than treatment for symptomatic malaria). We measured the impact of SMC and TB31F separately and in combination, in terms of the number of clinical cases prevented over this period (years 0–3), matching the frequency of mass ITN campaigns in the region. We varied the age group targeted: school-aged children (5–15 years of age), all children (up to 15 years of age, excluding infants <6 months of age), and the whole community (excluding infants <6 months of age). For all scenarios, we assumed a coverage of 80% in the targeted age group for both SMC and TB31F.

## RESULTS

**Figure 1.**
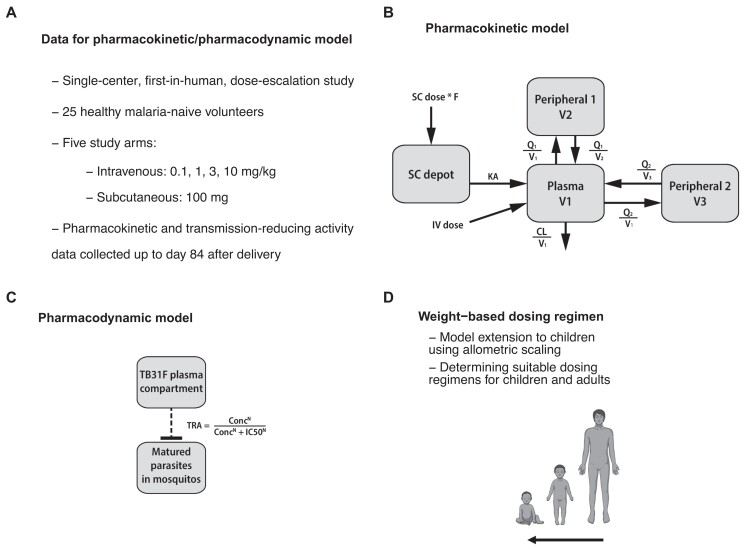
Overview of the pharmacokinetic/pharmacodynamic modeling workflow. *A*, We used data from the first-in-human trial of TB31F [14] for pharmacokinetic/pharmacodynamic modeling. *B*, The final pharmacokinetic model consisted of 3 disposition compartments and an absorption compartment for subcutaneously administered doses. *C*, The pharmacodynamic model described the relationship between TB31F concentrations and transmission-reducing activity (TRA). *D*, By extrapolating the pharmacokinetic model from adults to children by allometric scaling, we proposed a weight-based dosing regimen that is predicted to obtain a similar duration of effective TRA in children >6 mo of age compared to adults. Abbreviations: CL, clearance; Conc, TB31F concentration in the central pharmacokinetic compartment; IC_50_, TB31F concentration obtaining 50% transmission-reducing activity; IV, intravenous; KA, absorption constant; N, Hill factor; Q, intercompartmental clearance; SC, subcutaneous; TRA, transmission-reducing activity; V, volume.

**Figure 2.**
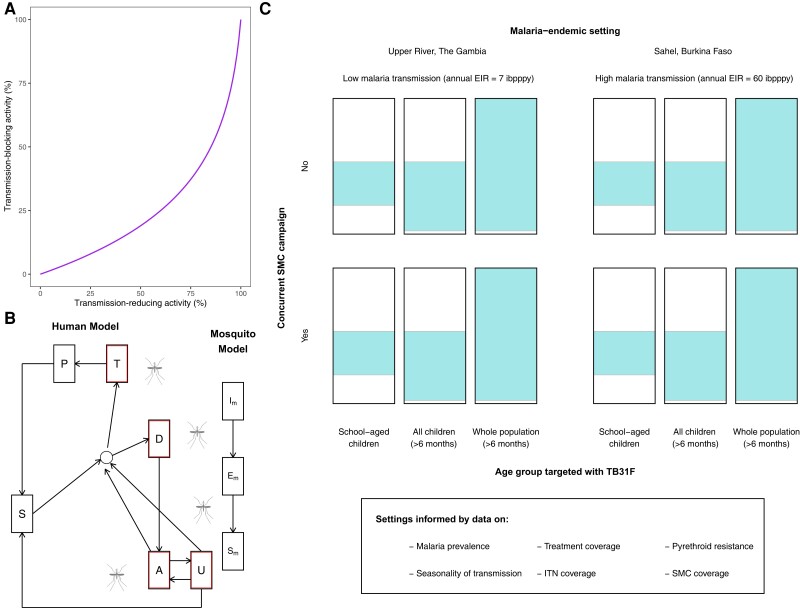
Overview of the epidemiological modeling. *A*, Predicted efficacy of TB31F in blocking malaria transmission events in the field. We use previous modeling work [23] to generate an estimated transmission-blocking activity for a given transmission-reducing activity, informed by data on oocyst counts found in naturally infected, wild-caught mosquitoes from Burkina Faso. *B*, Mathematical model of malaria transmission [21]. Individuals transition between 6 states: malaria-susceptible (S), untreated clinical disease (D), treated clinical disease (T), asymptomatic microscopy-detectable infection (A), asymptomatic submicroscopic infection (U), protected from malaria due to drug prophylaxis (P). Infected humans (outlined in red) can transmit malaria to mosquitoes. Mosquitoes can be susceptible to malaria (*S_M_*), infected but not yet infectious (*E_M_*), or infectious (*I_M_*). *C*, Modeled settings for the introduction of TB31F. We modeled the introduction of TB31F in 2 different seasonal malaria settings: a high-transmission site, based on the Sahel region of Burkina Faso, and a low-transmission site, based on the Upper River region of The Gambia. The values of the entomological inoculation rate shown are baseline values (ie, no interventions against malaria implemented). We vary the age group targeted with TB31F (the colored area of each rectangle indicates the proportion of the community targeted), administering the monoclonal antibody to school-aged children (5–15 y of age), all children aged >6 mo (0.5–15 y of age), and the whole community (excluding children <6 mo of age). The intervention is delivered alongside (or instead of) seasonal malaria chemoprevention for children aged 3–59 mo. Abbreviations: EIR, entomological inoculation rate; ibpppy, infectious bites per person per year; ITN, insecticide-treated bed nets; SMC, seasonal malaria chemoprevention.

We used data from the first-in-human TB31F trial (Figure 1*A*) to develop a model describing its pharmacokinetics (Figure 1*B*). A pharmacodynamic model was developed, describing the concentration-dependent TRA of TB31F, that is, the reduction in mosquito oocyst burden related to mAb concentration (Figure 1*C*). The pharmacokinetic model was extended to children, and a dosing regimen that obtains effective exposure over a period covering the malaria transmission season for most sub-Saharan African settings in both children and adults was identified (Figure 1*D*). TBA was calculated for simulated individuals using the predicted TRA (Figure 2*A*). A transmission model was used to explore different implementation strategies with TB31F in 12 epidemiological scenarios (Figure 2*B* and 2*C*).

### Pharmacokinetic/Pharmacodynamic Modeling

**Figure 3.**
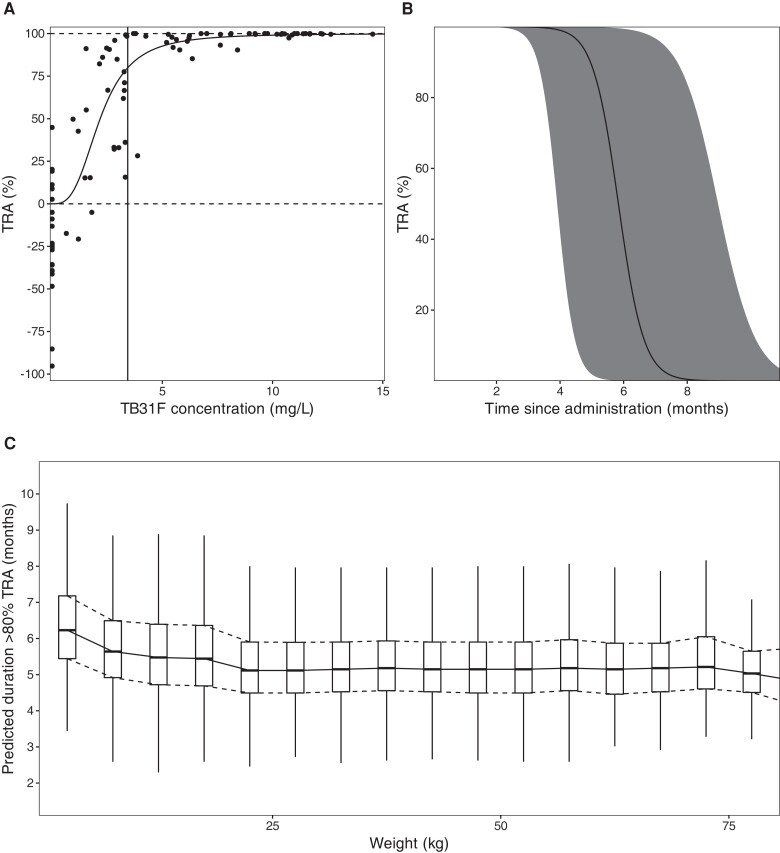
*A*, Model-predicted relationship between TB31F concentration and transmission-reducing activity (TRA). The solid line represents the model-predicted TRA and the dots represent observed TRA. The vertical line indicates the concentration resulting in 80% TRA (a TB31F concentration of 3.43 mg/L [95% CI, 3.34–3.53 mg/L]). *B*, Simulated TRA over time for all ages. The dose administered is 100 mg intravenously for individuals weighing <10 kg, 300 mg for 10–15 kg, and 700 mg for >15 kg. The continuous line represents the median and the band the 95% prediction interval. This regimen was used for in the following transmission modeling. *C*, The duration >80% TRA is predicted to be similar across all weights using the proposed dosing regimen. The boxes show the middle, upper, and lower quartiles per weight band with a 5-kg width. The whiskers depict the lowest and highest value within 1.5 times the interquartile range of the lower and upper quartiles, respectively. The simulations for *B* and *C* were performed with n = 114 000 virtual males and females aged ≥6 mo in equal proportions. Weights of these virtual individuals were simulated using a weight-for-age model appropriate for an African population. We used these demographics to extrapolate down to children 6 mo of age with weights representative for their age, which is especially important as the allometric scaling of clearance based on weight is age dependent.

Data from 275 pharmacokinetic samples of TB31F (from 24 individuals) and 3358 dissected mosquitoes from SMFA experiments were used in the pharmacokinetic/pharmacodynamic analyses (Figure 1*B*). The final parameters and goodness-of-fit plots are included in the Supplementary Materials (Supplementary Figures 1-5, Supplementary Table 1). TB31F pharmacokinetics were best described by a linear model with 3 disposition compartments and an absorption compartment for subcutaneous doses. The subcutaneous bioavailability fraction was estimated at 0.54 (95% confidence interval [CI], .45–.67). The mean absorption time was estimated at 3.2 days (95% CI, 2.8–4.2 days), which is in line with previously reported values for subcutaneous injection of other mAbs [27]. The relationship between TB31F concentration and TRA is shown in Figure 3*A*. The concentration achieving 80% TRA, historically used as threshold for potency [28], was 3.43 mg/L (95% CI, 3.34–3.53 mg/L). Given available data on the safety and efficacy of intravenous administration [14], we used this route of administration for our further analyses. We extrapolated the pharmacokinetic model to children and explored weight-based dosing, aiming to reach an equivalent duration >80% TRA in children as observed with 10 mg/kg in adults. With a single dose of 100 mg for individuals with weight ≤10 kg, 300 mg for individuals weighing 10–15 kg, and 700 mg for individuals weighing >15 kg, the median duration over which a mAb concentration associated with >80% TRA was sustained was >5 months (Figure 3*B*). This effective duration was comparable across all weights (Figure 3*C*); thus, this regimen was used in the simulations described from here on.

### Predicted Public Health Impact

**Figure 4.**
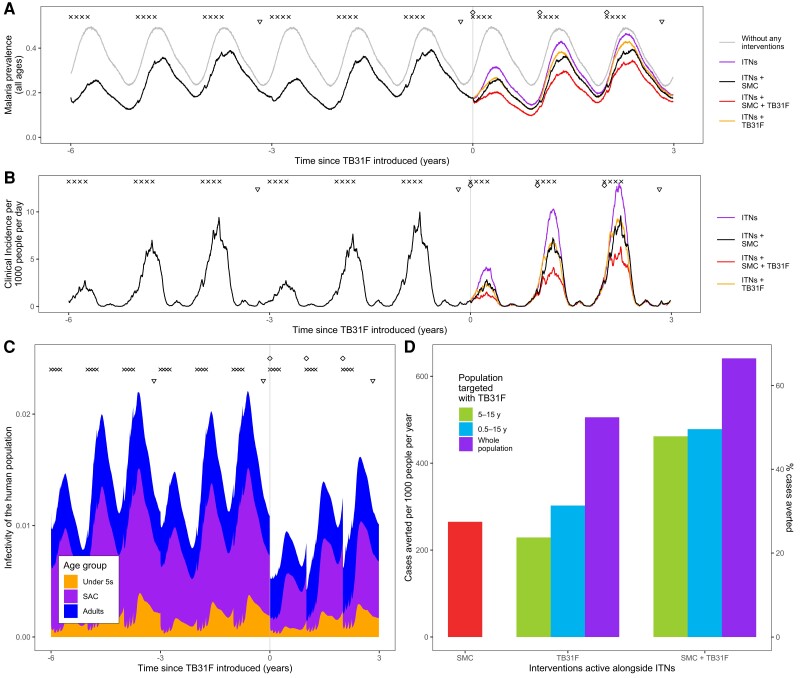
Modeling the introduction of TB31F intervention in the Sahel region of Burkina Faso (high-transmission setting). *A*, Malaria parasite prevalence (by microscopy) and changes over time as interventions additional to insecticide-treated nets (ITNs) are introduced. The symbols at the top of the plot indicate the timings of intervention delivery. We assume that ITN distribution campaigns (triangles) and seasonal malaria chemoprevention (SMC) delivery (crosses) have occurred regularly prior to the introduction of TB31F (diamonds) at time zero. The gray curve indicates malaria prevalence prior to the introduction of control interventions. The colored lines indicate the interventions used after time zero. SMC is delivered to children aged 3–59 mo at 80% coverage; in *A–C*, TB31F is delivered to school-aged children (SAC) (5–15 y of age) at 80% coverage. *B*, Model-derived estimates of clinical incidence in the population over time. The impact of TB31F is most apparent in the second and third years of rollout, as the new ITNs prevent a high proportion of cases in the first year. *C*, The changing infectivity of the human population to a blood-feeding mosquito as interventions are introduced. This panel shows how the infectious reservoir (not adjusted for age-dependent mosquito biting rates) of malaria is distributed among 3 age groups: children <5 y of age, SAC, and adults (>15 y of age). After TB31F is introduced to SAC, the infectiousness of this age group is greatly reduced for several months. *D*, Cases averted due to SMC and TB31F interventions. Over a 3-year period, we measure the reduction in cases on top of those prevented by ITNs. Here we vary the age group targeted with TB31F (SAC, all children aged >6 mo, and all age groups aged >6 mo in the community), assuming a coverage of 80% within the target age group. We quantify the clinical cases averted in 2 ways: the number of cases averted per 1000 people per year (left y-axis) and the percentage of the total number cases that is averted (right y-axis). In terms of cases averted per dose of TB31F administered, targeting children aged 5–15 y proved more efficient than targeting children aged 0.5–15 y, or the whole population aged >6 mo (Supplementary Table 2).

**Figure 5.**
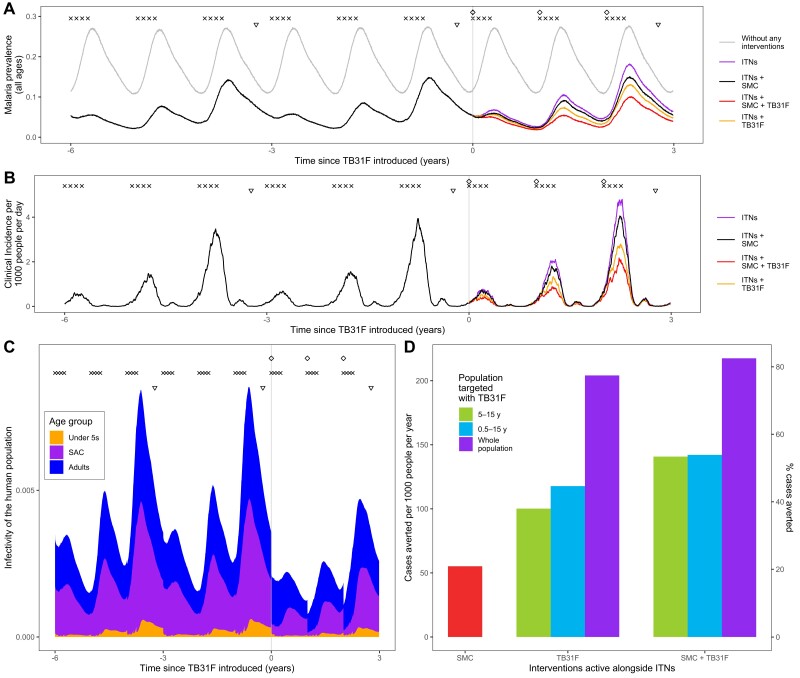
Modeling the introduction of TB31F intervention in the Upper River region of the Gambia (low-transmission setting). *A*, Modeled malaria parasite prevalence (by microscopy) is reduced as additional interventions to insecticide-treated nets (ITNs) are introduced. The symbols at the top of the plot indicate the timings of intervention delivery. We assume that ITN distribution campaigns (triangles) and seasonal malaria chemoprevention (SMC) delivery (crosses) have occurred regularly prior to the introduction of TB31F (diamonds). The gray curve indicates malaria prevalence prior to the introduction of control interventions. ITNs, distributed every 3 years, have the largest impact on prevalence in the first year after distribution. SMC is delivered to children aged 3–59 mo at 80% coverage; in *A* and *B*, TB31F is delivered to school-aged children (SAC) (5–15 y of age) at 80% coverage. *B*, Modeled clinical incidence in the population over time. The impact of TB31F is most apparent in the second and third years of rollout, as the new ITNs prevent a high proportion of cases in the first year. *C*, Infectivity of the human population, as interventions are introduced. This panel shows how the infectious reservoir (not adjusted for age-dependent mosquito biting rates) of malaria is distributed among 3 age groups: children aged <5 y, SAC, and adults (>15 y of age). After TB31F is introduced to SAC, the infectiousness of this age group is greatly reduced for several months. *D*, Cases averted due to SMC and TB31F. Over a 3-year period, we measure the reduction in cases on top of those prevented by ITNs. Here we vary the age group targeted with TB31F (SAC, all children aged >6 mo, and all age groups >6 mo in the community), assuming a coverage of 80% within the target age group. We quantify the clinical cases averted in 2 ways: the number of cases averted per 1000 people per year, and the percentage of the total number of clinical cases averted (right y-axis). In terms of cases averted per dose of TB31F administered, targeting SAC proved more efficient than targeting children aged 0.5–15 y, or the whole population >6 mo of age (Supplementary Table 2).

A standalone implementation of the pharmacokinetic model including interindividual variation, a weight-for-age model, and weight-based dosing was developed. It was incorporated into a model of *P falciparum* malaria transmission, in order to predict the impact that TB31F could have in 2 settings where malaria transmission is highly seasonal. These settings were tailored to resemble malaria transmission in the high-transmission Sahel region in Burkina Faso (Figure 4) and the Upper River region of the Gambia, where malaria transmission is considerably lower (Figure 5). In both settings, ITNs are distributed every 3 years, and children aged 3–59 months receive SMC during the transmission season (Figures 4*A* and 5*A*). We simulated the impact of annual administration of TB31F, with a coverage of 80% of the target age group, for 3 consecutive years. To compare SMC and TB31F, SMC was withdrawn in our baseline scenario, leaving ITNs as the only active public health intervention. Although we are not proposing that TB31F could replace SMC in malaria-endemic settings, withdrawing SMC in our simulations enables a comparison of the community-wide impact of these interventions to be made.

In both malaria-endemic settings (Figures 4 and 5), TB31F was predicted to have a pronounced impact on clinical incidence across the community. In the high-transmission setting, administering TB31F to school-aged children (5–15 years old) has a similar impact as delivering SMC to young children (3–59 months). However, a much larger impact is seen when the interventions are delivered in tandem. In this region of Burkina Faso, delivering TB31F to school-aged children in addition to delivering SMC to young children (3–59 months) is predicted to avert 48% of all clinical cases (462 cases per 1000 people per year) over a 3-year period (Figure 4*D*), compared to the counterfactual scenario in which ITNs are the only active intervention. This value rises to 66% of cases (641 cases per 1000 people per year), if TB31F were administered to all age groups (Figure 3*D*). In the low-transmission setting, administering TB31F to school-aged children had a larger impact than delivering SMC to children aged 3–59 months. As in the high-transmission setting, delivering the 2 interventions in combination had a large impact: Administering TB31F to school-aged children and also delivering SMC to children aged 3–59 months led to 53% of clinical cases (141 cases per 1000 people per year) being averted. This value rose to 82% (217 cases per 1000 people per year) when the TB31F campaign was extended to all age groups in the community. In both settings, targeting school-aged children with TB31F was the most efficient intervention in terms of cases averted per dose administered, but broadening the target population to also include other age groups substantially increased the overall impact of the intervention. The cases averted per dose of TB31F administered will vary depending on whether TB31F is delivered instead of or alongside SMC (Supplementary Table 2). Moreover, if coverage of SMC in children aged <5 years is high, there is little additional value in targeting this age group with a transmission-blocking intervention, especially in low-transmission settings (Figure 5*D*, Supplementary Table 2).

**Figure 6.**
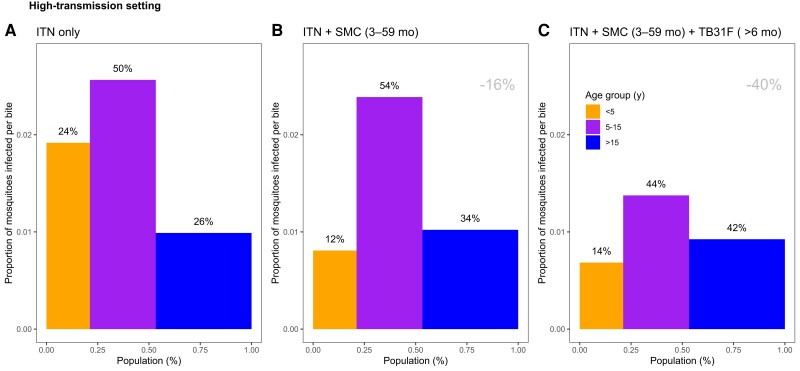
The changing infectious reservoir of malaria in a high-transmission setting, as interventions are introduced. Model-derived projections of how different public health interventions against malaria influence the magnitude and composition of the human infectious reservoir. We show contributions to the infectious reservoir from 3 age groups in a high-transmission setting (based on the Sahel region of Burkina Faso, as used in Figure 4): children <5 years of age, school-aged children (5–15 y of age), and adults. These contributions are influenced by the average per-person infectivity (y-axes) and the relative sizes of the 3 subpopulations (x-axes). We first assessed the infectious reservoir when the only intervention in use is insecticide-treated nets (*A*). We then assessed the impact of delivering seasonal malaria chemoprevention (SMC) to 80% of children aged 3–59 mo (*B*). Third, we measured the impact of delivering TB13F to 80% of school-aged children (*C*). In this setting, the introduction of TB31F reduces the infectivity in all age groups, due to a reduction in malaria transmission. It also results in a shift in the relative importance of age groups for the infectious reservoir, increasing the contribution that adults make to malaria transmission. The results in each panel were generated by averaging over a 3-year period. *A* corresponds to a time period prior to the introduction of SMC. *B* corresponds to the 3-year period (−3, 0) in Figure 4*C*; *C* corresponds to the 3-year period (0, 3) in Figure 4*C*. The percentages above each bar indicate the contribution that each age group makes to the infectious reservoir. The percentage reductions (gray text, top-right corner of *B* and *C*) indicate the overall reduction in the infectious reservoir relative to the insecticide-treated net (ITN)–only scenario. To allow a direct comparison between this setting and the low-transmission setting (Figure 7), we use a generic demography for sub-Saharan Africa [21]. In Supplementary Figure 6, we repeat the analysis for the case where TB31F was administered to all age groups. Note that the results presented here are not adjusted for age-dependent biting of mosquitoes.

**Figure 7.**
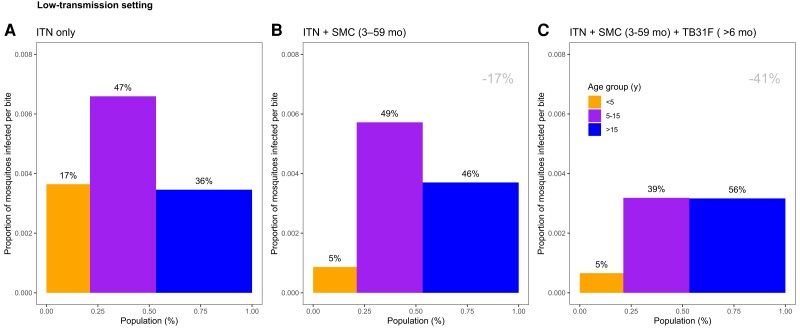
The changing infectious reservoir of malaria in a low-transmission setting, as interventions are introduced. Model-derived projections of how different public health interventions against malaria influence the magnitude and composition of the human infectious reservoir. We show contributions to the infectious reservoir from 3 age groups in a low-transmission setting (based on the Upper River region of the Gambia, as used in Figure 5): children <5 years of age, school-aged children (5–15 y of age), and adults. These contributions are influenced by the average per-person infectivity (y-axes), and the relative sizes of the 3 subpopulations (x-axes). We first assessed the infectious reservoir when the only intervention in use is insecticide-treated nets (*A*). We then assessed the impact of delivering seasonal malaria chemoprevention (SMC) to 80% of children aged 3–59 mo (*B*). Third, we measured the impact of delivering TB13F to 80% of school-aged children (*C*). In this setting, the introduction of TB31F reduces the infectivity in all age groups, due to a reduction in malaria transmission. It also results in a shift in the relative importance of age groups for the infectious reservoir, increasing the contribution that adults make to malaria transmission. The results in each panel were generated by averaging over a 3-year period. *A* corresponds to a time period prior to the introduction of SMC. *B* corresponds to the 3-year period (−3, 0) in Figure 4*C*; *C* corresponds to the 3-year period (0, 3) in Figure 4*C*. The percentages above each bar indicates the contribution that each age group makes to the infectious reservoir. The percentage reductions (gray text, top-right corner of *B* and *C*) indicate the overall reduction in the infectious reservoir relative to the insecticide-treated net (ITN)–only scenario. To allow a direct comparison between this setting and the high-transmission setting (Figure 6), we use a generic demography for sub-Saharan Africa [21]. In Supplementary Figure 6, we repeat the analysis for the case where TB31F was administered to all age groups. Note that the results presented here are not adjusted for age-dependent biting of mosquitoes.

The composition of the infectious reservoir changes when SMC and TB31F are introduced. Figures 4*C* and 5*C* show how the contribution of different populations to transmission changes over time when SMC and SMC plus TB31F are implemented. In both the high-transmission (Figure 6) and low-transmission (Figure 7) sites, school-aged children make an important contribution to the infectious reservoir (50% in the high-transmission site; 47% in the low-transmission site) prior to the implementation of SMC and TB31F. Compared to the high-transmission setting, children aged <5 years make a smaller contribution to the infectious reservoir in the low-transmission setting. When TB31F is administered to 80% of school-aged children alongside standard implementations of ITN and SMC, the overall infectious reservoir shrinks (by 29% in the high-transmission site and by 26% in the low-transmission site), and the relative contribution of adults to malaria transmission increases. In Supplementary Figure 6, we show the impact of delivering TB31F to 80% of the population in each setting (excluding children <6 months of age), causing a larger reduction in community-level infectivity. Two sensitivity analyses were performed to assess the robustness of our results to changes in the TRA–TBA relationship (Supplementary Figure 7), the clearance rate of the mAb (Supplementary Figure 8), and the relationship between TRA and antibody concentration (Supplementary Figure 9). These analyses are discussed in more detail in the Supplementary Results. We also outline how the public health impact of TB31F could be measured within a cluster-randomized trial. Taking malaria prevalence as trial endpoint, we use transmission modeling to estimate the effect size and guide sample size calculations for the hypothesized trial (Supplementary Figure 10). In the Supplementary Results, we touch upon several important factors to consider when aiming to measure the impact of a transmission-blocking intervention in field settings.

## DISCUSSION

In this work, we have combined pharmacokinetic/pharmacodynamic modeling with an established malaria transmission model. We found that annual administration of a single weight-based dose of TB31F at the start of the malaria season resulted in a substantial reduction in malaria transmission and clinical incidence. Of the 3 age groups considered, targeting school-aged children had the largest per-dose impact. However, the total impact of the intervention was substantially increased when the other age groups were also targeted and when the mAb intervention was combined with SMC.

Interventions that specifically target the transmission of malaria differ from conventional malaria control measures by providing a delayed, rather than direct, benefit [8]. While they have clear and measurable biological endpoints in the reduction in mosquito infections, predicting their corresponding public health benefit is complex. Furthermore, the eligible population for transmission-blocking interventions is broader than that of, for example, SMC, which primarily reduces malaria incidence in young children who are also the intervention recipients. Importantly, the age groups that drive malaria transmission are not those most at risk of severe disease [17, 29, 30]. Recently, TB31F was tested in healthy adult volunteers where it had an excellent safety profile, including for the dose considered here. Postinfusion antibody kinetics and potency indicated prolonged transmission-blocking efficacy [14]. We translated TB31F's pharmacokinetic properties in Western adult volunteers to a sub-Saharan African population of both children and adults, incorporating allometric scaling of pharmacokinetic parameters using body weight. We identified a simple dosing regimen to facilitate implementation. With this dosing strategy, we predict that >80% TRA for a median duration of 5 months can be achieved.

We next simulated the introduction of TB31F into malaria-endemic settings. When targeting the entire population (excluding children <6 months of age) with TB31F at 80% coverage on top of ITNs and SMC, a single annual administration reduced clinical malaria incidence by 54% and 74% over 3 years in high- and low-transmission settings, respectively. While the overall impact of TB31F decreases when only certain age groups are targeted, the uneven contribution of different populations to onward transmission [17, 29, 31] allows demographic targeting to increase intervention efficiency. Choosing age groups to prioritize with a transmission-blocking intervention relies on understanding who contributes most to malaria transmission in a particular setting. Our work demonstrates that the impact of administering TB31F to a subset of the population, namely school-aged children (5–15 years of age), may achieve a comparable impact on clinical cases as SMC (given to children aged 3–59 months) in a high-transmission setting and that the impact may even exceed that of SMC in a low-transmission setting. While we do not suggest that SMC be withheld from populations in sub-Saharan African regions, where it achieves a very high level of personal protection against clinical malaria [32], our findings demonstrate the large public health impact a malaria transmission-blocking intervention can have. Our findings further indicate that combining SMC and TB31F may considerably increase the number of cases averted. A recent clinical trial from Mali demonstrated that combining the preerythrocytic malaria vaccine RTS,S with SMC was superior to either intervention delivered alone [33]. Our findings further highlight that interventions against malaria will reshape the human infectious reservoir, requiring a periodic review of strategies to maximize intervention impact.

It is significant that only a single dose of TB31F is needed to achieve prolonged activity. This facilitates implementation on a large scale; a less potent mAb would require multiple doses per year or antibody half-life extension to achieve the same effective duration. A potent malaria mAb (CIS43LS) was engineered to approximately double the half-life and has induced strong anti-infection activity in human volunteers [34, 35]. Similar half-life extension technologies could also be of value for TB31F [35–37] and would lower the cost of the intervention by lowering the administered dose and may extend its effective period further. Subcutaneous administration is currently limited by a maximum volume that can be administered at a single injection site (100 mg was administered spread over 2 injection sites in the TB31F trial), and the currently suggested dosages may not yet be practical in subcutaneous administration. By extending the half-life, it would be possible to achieve >80% TRA for a longer duration with a similar injection volume. Although intravenous administration of a malaria vaccine has been achieved at a large scale [38], subcutaneous administration would have considerable operational advantages.

Coadministration of a transmission-blocking intervention and preerythrocytic vaccine has already shown a synergistic potential [39]. Combining the anti-infection mAb CIS43LS with the transmission-blocking mAb TB31F holds considerable promise. Importantly, TB31F may also prevent the spread of mutant parasites that escape CIS43LS. TB31F targets Pfs48/45, which has limited genetic variation, a shared advantage of many transmission-blocking vaccine candidates [10]. The potency of TB31F has been demonstrated against 2 genetically distant parasite lines [14]; the original rat mAb 85RF45.1 that formed the basis for TB31F has been tested against genetically diverse gametocyte isolates from Cameroon and Burkina Faso, with no indications for escape mutants [40].

Our study has a number of limitations. The pharmacokinetic extrapolation from healthy Western adults to African populations has a solid mechanistic and empirical foundation [41, 42], but dedicated studies of TB31F in African populations, including children, are needed to confirm its safety and could test TB31F formulations for either intravenous or subcutaneous administration. Furthermore, the safety, and risk-benefit ratio of administering a transmission-blocking mAb to pregnant women should be carefully assessed. As the transmission model contains no spatial structure, the mAb-derived benefit is shared equally between those who received the intervention and those who did not. This may not always be the case, for example, if certain households have very high intervention coverage and malaria transmission is highly focal. This could have implications for clinical trials of transmission-blocking interventions that measure epidemiological endpoints. It may be that the preferred use case of mAb would be to try and push the disease to elimination in low-transmission settings. Predicting this would require an understanding of the changing patterns of intervention use, the ability of local health systems to treat and diagnose cases, and the level of disease importation. These factors should be considered in further work.

We have shown that TB31F may be an effective intervention against malaria in settings with a well-defined malaria season. Targeting children 5–15 years old proved most efficient for reducing malaria transmission. If TB31F is implemented alongside SMC, we predict an even larger impact. A transmission-blocking mAb used alongside established interventions could play a crucial role in decreasing the global burden of malaria.

## Supplementary Data

Supplementary materials are available at *The Journal of Infectious Diseases* online. Consisting of data provided by the authors to benefit the reader, the posted materials are not copyedited and are the sole responsibility of the authors, so questions or comments should be addressed to the corresponding author.

## Supplementary Material

jiad101_Supplementary_Data

## Notes

***Acknowledgments.*** We thank the participants and study personnel of the first-in-human trial. We are grateful to Dr Elin Svensson from Uppsala University and Radboud University Medical Center for her advice regarding the pharmacokinetic/pharmacodynamic modeling analysis of this study.

***Ethics statement*.** All the data analysis conducted during this research was secondary and used studies that had obtained ethical approval previously from the appropriate organizations. Patients or the public were not involved in the design, conduct, reporting, or dissemination plans of our research.

***Financial support***. This study was supported by a fellowship from the European Research Council to T. B. (ERC-CoG 864180; QUANTUM) and PATH's Malaria Vaccine Initiative. J. D. C., G. D. C., and T. S. C. acknowledge funding from the Medical Research Council (MRC) Centre for Global Infectious Disease Analysis (reference number MR/R015600/1), jointly funded by the MRC and the UK Foreign, Commonwealth and Development Office (FCDO), under the MRC/FCDO Concordat agreement, and is also part of the EDCTP2 program supported by the European Union. G. D. C. acknowledges funding from the Wellcome Trust (reference number 220900/Z/20/Z). Funding to pay the Open Access publication charges for this article was provided by Imperial College London, under a Read and Publish agreement.
