# Supplementary material for: Modeling the Impact of a Highly Potent Plasmodium falciparum Transmission-Blocking Monoclonal Antibody in Areas of Seasonal Malaria Transmission
Source: J Infect Dis. 2023 Apr 12;228(2):212–23. doi: 10.1093/infdis/jiad101 (PMC10345482; doi:10.1093/infdis/jiad101)
Supplement: jiad101_Supplementary_Data [file jiad101_supplementary_data.pdf]

Modeling the impact of a highly potent Plasmodium falciparum transmission-blocking monoclonal antibody in areas of seasonal malaria transmission

Authors
Joseph D Challenger[1]§*, Stijn W van Beek[2]§, Rob ter Heine[2], Saskia C van der Boor[3], Giovanni D Charles[1], Merel J Smit[3], Chris Ockenhouse[4], John J Aponte[5], Matthew BB McCall[3], Matthijs M Jore[3], Thomas S Churcher[1]§, Teun Bousema[3]§
§ Contributed equally
*Corresponding author

Affiliations
1 Medical Research Council Centre for Global Infections Disease Analysis, Department of Infectious Disease Epidemiology, Imperial College London, London, United Kingdom
2 Department of Pharmacy, Radboud Institute for Health Sciences, Radboud University Medical Center, Nijmegen, The Netherlands
3 Department of Medical Microbiology, Radboud University Medical Center, Nijmegen, the Netherlands
4 PATH Center for Vaccine Innovation and Access (CVIA), Washington DC, USA
5 PATH Center for Vaccine Innovation and Access (CVIA), Geneva, Switzerland

SUPPLEMENTARY MATERIALS

This document contains:

1. **Supplementary Methods**
2. **Supplemntary Results**
3. **Supplementary References**
4. **Supplementary Tables 1 & 2**
5. **Supplementary Figures 1-10**
6. **NONMEM code for the final pharmacokinetic model**

References 1-42 are numbered as per the main text.

Supplementary Methods

Pharmacokinetic/pharmacodynamic modelling

Parametric nonlinear mixed-effects population modelling was performed to analyse the pharmacokinetic data and the relationship with transmission-reducing activity (43). Population models are comprised of a structural fixed-effects model and a stochastic random-effects model (44). The structural model describes the fixed effects using differential equations. The stochastic model describes the random effects of variability and residual error. NONMEM version 7.4.1 was used to perform the population modelling (20). Allometric scaling to a total body weight of 70 kg for volume, clearance and inter-compartmental flow parameters was included during parameter estimation from the start of model development to account for differences in weight, with exponents of respectively 1 and 0.75 for volume and clearance parameters. Different numbers of disposition compartments were explored to describe the pharmacokinetics of TB31F. Subcutaneous absorption was described using a dose depot and first-order absorption into the central compartment. Inter-individual variability was explored on the volume and clearance parameters of the pharmacokinetic model.

The pharmacodynamic model was developed following the pharmacokinetic model in a sequential analysis, using individual pharmacokinetic parameter estimates as input (45). The relationship between TB31F concentrations and transmission-reducing activity was modelled using oocyst count data of individual mosquitos as analysed in the standard membrane feeding assay (SMFA). In the SMFA, the number of oocysts per mosquito was determined for 20 mosquitos per pharmacokinetic blood sample The distribution of oocyst counts was described by a negative binomial distribution function for which mean baseline oocyst count and overdispersion were estimated. Inter-assay variability was included on the mean baseline oocyst count. An Emax model was used to characterise the relationship between TB31F concentrations and reduction in oocyst count (TRA) using the following equation:

$$TRA = \frac{C^N}{C^N + IC50^N}.$$

Here TRA is the proportion reducing the mean baseline count of the negative binomial distribution, C is the TB31F concentration in the central pharmacokinetic compartment, N is the Hill factor and IC50 is the concentration at which 50% effect is observed. In the clinical trial (14), TB31F concentration in serum was expressed and related to transmission reducing activity when this serum was mixed with gametocyte infected red blood cells and a source of complement. In this work, we adhered to this approach to ensure TB31F antibody concentrations were reliably associated with transmission-reducing activity as observed in the clinical trial.

The pharmacokinetic model parameters were estimated using the first-order conditional estimation method with interaction and the pharmacodynamic parameters using the Laplacian estimation method. The inter-individual and inter-assay parameters in the pharmacokinetic and pharmacodynamic models were assumed to be log-normally distributed. Residual variability in TB31F pharmacokinetics was implemented using a proportional error model. The uncertainty of the pharmacokinetic and pharmacodynamic parameters was determined using sampling importance resampling (46). Model selection and evaluation was based on goodness-of-fit plots and comparison of the objective function value. A change in objective function value of 3.84 points between two nested models, associated with a significance level of 0.05, was considered statistically significant. Non-nested models were compared using the Akaike Information Criterion.

We developed a practical weight-based intravenous dosing regimen reaching an equivalent duration >80% TRA in children as observed with the highest dose tested in adults using simulations with the

pharmacokinetic/pharmacodynamic model. The pharmacokinetic/pharmacodynamic model was extended from adults to children by implementing an age-dependent exponent for allometric scaling of pharmacokinetic clearance parameters (41). An exponent of 0.75 was used from the age of 5 years onwards. For younger children 2 to 5 years and 6 months to 2 years of age, exponents of 0.9 and 1.0 were used, respectively. Regarding the extrapolation of pharmacokinetics from adults to children, the correctness of our assumptions should certainly be prospectively evaluated. However, this has no impact on the conclusions in this work as it would only result in a different dose being selected. This is because the dose was determined based on the duration of its effect. A different allometric relationship would thus result in a different dose, but the same activity. We simulated female and male age groups of 6 months and every year from 1 to 18 years. Every group included 5000 virtual individuals. Weight data was simulated as described in the next Supplemental Methods section. Weight-based dosing regimens were explored aiming to reach a time to 80% transmission-reducing effect similar to a 70 kg adult administered 10 mg/kg TB31F intravenously.

Weight-for-age modelling methods

Weight data was simulated following a previously described method using LMS data (24). This method uses reference values for weight by sex and age for children provided by the World Health Organization and Centers for Disease Control and Prevention growth charts (47,48). These growth charts allow calculation of the individual weight-for-age z-score, a measure of how many standard deviations the weight of a child differs from the median given their sex and age. This score is determined by sex- and age-specific parameters for the Box-Cox transformation (L), median (M) and coefficient of variation (S) which are reported in the growth charts. The z-score comes from a normal distribution with a mean of zero and standard deviation of one. Following this distribution, the weight of children may also be simulated using these charts. While these growth charts describe optimal development of children, Wasmann et al. additionally describe a correction accounting for stunted growth allowing more reliable simulation of an African population. A more detailed description of this workflow and the simulation script can be found in the original publication (24). As the charts stop at 18 years, simulation of adults was not incorporated in the workflow and we assumed the distribution at 18 years for all adults. This assumption will not hold in a western population, but is expected to be reliable in an African population.

Predicting public health impact using transmission modelling

In order to assess the potential utility of TB31F as a public health intervention, we have incorporated it into a stochastic, individual-based transmission model. This transmission model is described in detail elsewhere (21,22). Briefly, the malaria status of humans can transition between 6 states (Figure 1, panel 7). When uninfected individuals (*S*) are infected by a feeding Anopheles mosquito, the infection can either become symptomatic or remain asymptomatic (*A*), depending on their degree of naturally acquired immunity against malaria. Symptomatic infections are either treated with antimalarials (*T*) or remain untreated (*D*), which will depend on the level of treatment coverage selected in the model. If the infection is treated, the individual benefits from a period of prophylaxis (*P*), during which they are protected against further infectious bites. The individual then returns to being malaria-susceptible (*S*). If the symptomatic infection is untreated, the infection will become a long-lasting asymptomatic infection (*A*). Eventually, the parasite densities in these infections will be reduced due to the human immune response, and the infection becomes undetectable by slide microscopy (*U*). When the infection is cleared by the immune response, the individual returns to being malaria-susceptible (*S*). Humans in states *D, T, A,* and *U* are able to transmit malaria to feeding mosquitos, although not all infected humans are equally infectious, those with sub-microscopic infections having the lowest per-bite probability of infecting a mosquito. The mosquito component of the model is simpler: mosquitoes are either susceptible to malaria (S_M), infected but not yet

infectious (E_M), or infectious (I_M). The size of the adult female mosquito population, relative to that of the human population, determines the baseline intensity of transmission in the model.

The introduction of TB31F into two sites in west Africa is investigated, one of low malaria transmission, the other high. Using rainfall data, we chose two sites for which malaria transmission is highly seasonal. The high-transmission site was characterised to be representative of the Sahel region of Burkina Faso. For the rainfall patterns utilised for this region in the transmission model (25), 82% of the modelled annual clinical incidence of malaria occurs within 4 consecutive months. The low-transmission site was based on the Upper River region of the Gambia with 84% of the modelled annual clinical incidence of malaria occurs within 4 consecutive months. Before modelling the introduction of TB31F, we calibrated the model to reproduce the level of endemicity observed within each region according to the effectiveness of antimalarial interventions in use in the region. The level of endemicity was represented by estimates of the prevalence of falciparum malaria in 2-10 year olds ($PfPR_{2\text{-}10}$) for the administrative unit 1 region from the Malaria Atlas Project (MAP) (26). To link transmission intensity to malaria prevalence, it is important to account for public health interventions currently in place. For these sites, we utilised data on ITN usage (60% in each setting, data from MAP (26)), treatment coverage for symptomatic malaria (50% in both settings, data from MAP (26)), and the coverage of SMC, which is administered as 4 rounds of sulfadoxine–pyrimethamine plus amodiaquine a year to children aged 3-59 months during the malaria season (80% in the Sahel region of Burkina Faso, 60% in the Upper River region of the Gambia, data from latest World Malaria Report (1)). For all MAP data utilised here, we obtained the latest estimates available through their website, as of January 2022 (all data pertain to 2019) (https://malariaatlas.org, accessed January 10th 2022). The effectiveness of ITNs also depends on the levels of pyrethroid resistance observed in wild Anopheles mosquitoes in these regions (49), as this will inform the lethality of the ITNs used in the areas against the mosquitoes (50). In both regions it is assumed that all nets are pyrethroid-piperonyl butoxide ITNs and are distributed every three years in mass campaigns. For simplicity it is assumed each mass campaign achieves the same level of ITN usage (as defined above), after which usage and ITN effectiveness declines at realistic rates (50). For the simulations of the high-transmission site, we simulated a population of 25,000. For the low-transmission site, we increased the population size to 50,000, as the lower incidence of malaria led to larger between-simulation variation. For the public health projections, we ran 50 simulations and reported the mean impact of TB31F and SMC.

An important consideration in the modelling presented here is the relationship between TRA and TBA, which is governed by the parasite load in naturally infected, wild mosquitoes. The higher the parasite load, the harder it is for a transmission-blocking intervention to completely block the development of parasites within the mosquito (51). As outlined by Challenger et al. (23), we use a mathematical relationship between TRA and TBA. This relationship depends on the distribution of oocyst counts in naturally infected mosquitoes, which is described by a zero-truncated negative binomial distribution. The distribution is characterised by two parameters: *m* (which would be the mean of the distribution, were it not truncated) and *k* (the dispersion parameter). The relationship between TRA and TBA takes the following form (see Challenger et al. (23) for the derivation):

$$TBA(TRA) = \frac{1}{1-\left(\frac{k}{k+m}\right)^k}\left[\left(\frac{k}{k+m(1-TRA)}\right)^k - \left(\frac{k}{k+m}\right)^k\right].$$

We used oocyst counts measured in naturally infected mosquitoes in Burkina Faso (52) to inform values of *m* and *k* Informed by the Burkina Faso data, we used parameter values *m*=0.000157 and *k*=0.00000495, which results in a mean oocyst count of 9.1. Note that here we fit one zero-truncated negative binomial distribution to the data: in Ref. (23) a more complex model, which assumes different TRA-TBA models for different malaria infections (e.g. symptomatic versus asymptomatic malaria), was also fitted. Here we choose the simpler approach. Data on oocyst counts in wild mosquitoes are rarely reported and may vary across settings. As a sensitivity analysis, we repeated

the analyses used to generate Figures 4 and 5 whilst varying the mean oocyst count. We generated results for an adjusted TRA-TBA relationship, obtained by first quartering (m=0.0000158, mean=2.2), then doubling (m=0.000398, mean=18.2), the mean number of oocysts, holding the dispersion parameter fixed. The results of the sensitivity analysis are shown in Supplementary Figure 7, along with a plot of the modified TRA-TBA relationships.

Supplementary Results

Predicted public health impact

An important factor in the predicted impact of a transmission-blocking intervention is the relationship between TRA and TBA (Methods). The relationship used here is informed by detailed data on naturally infected mosquitoes, collected in a high-transmission setting in Burkina Faso in 2014 (52). Parasite loads may vary between settings, e.g. due to transmission intensity, or vector composition. As detailed data such as those reported by Bompard et al. (52) are not often reported, we have used the same relationship between TBA and TRA for both sites examined here. In Supplementary Figure 7 we show the influence of parasite load on our modelling projections, by quartering and doubling the mean oocyst counts. We find that decreasing the mean oocyst counts does lead to a slightly larger public health impact, and *vice versa*. But our results are quite robust to changes in the TRA-TBA relationship, which has not been widely characterised *in natura*.

We also examined the potential impact of faster clearance of the mAb, which could be expected in the translation from the European trial participants to individuals living in malaria-endemic countries (Supplementary Figure 8). Studies reporting on the effect of cachexia describe a clearance that is around 1.33-fold higher (53–55). We find that a 1.33-fold increase in the clearance parameter in the pharmacokinetic model reduces the typical amount of time an individual maintains TRA>80% (Supplementary Figure 8A). This would reduce the public health impact of TB31F, but the modelling predicts that this reduction would be small (Supplementary Figures 8B & 8C). This is because the two settings selected here are highly seasonal: we expect that increasing the clearance would have a larger impact in settings with a longer transmission season, or malaria transmission throughout the year.

The relationship between TRA and antibody concentration, and its impact on our public health predictions is also interesting to explore. We have high confidence in the fitted relationship obtained here, due to the narrow confidence intervals in the parameter estimates of IC50 and the Hill parameter (Supplementary Table 1). However, we can also show that our modelling results are robust to much larger increases in the IC50. In Supplementary Figure 9A we show the impact that increasing the value of IC50 by 50% and 100% has on the TRA. In Supplementary Figure 9B we combine this with the pharmacokinetic model, which indicates that increasing IC50 in this way only slightly reduces the duration of TRA>80%. This reduction is much smaller than that obtained by increasing the clearance rate for the mAb (Supplementary Figure 8B). Therefore, we can be confident that our modelling projections for the public health impact of TB31F are robust to changes in this relationship.

Finally, we outline how the public health impact of TB31F could be measured in a trial setting. Due to the community-level benefit that this monoclonal antibody provides, we have utilised a cluster randomised design in this hypothetical scenario. We consider the same low-transmission setting considered in this analysis, based on the Upper River region of the Gambia (Figure 5). In the cluster randomised clinical trial (CRCT), clusters are randomised to receive either TB31F or a placebo. In all clusters ITNs are provided and children aged 3-59 months receive SMC (coverage levels at the same levels as considered for the scenarios modelled here). We assume that TB31F is administered to all

age groups (excluding children under 6 months), at 80% coverage. Supplementary Figure 10A shows the prevalence of malaria in the population over time. Note that this panel differs from Figure 5A, since in this case TB31F is administered to all age groups (excluding children under 6 months), rather than only children aged 5-15 years. The trial is carried out over a two-year period, with TB31F administered at the start of each malaria season. At the end of the transmission season in the second year, malaria prevalence is measured in each cluster, by cross-sectional survey (dashed vertical line in Supplementary Figure 10A). The transmission modelling predicts that by this time malaria prevalence should have dropped from 0.14 to 0.04. However, it may not be feasible to measure the full benefit in a trial setting. The transmission model describes a ‘closed’ human population i.e. there is no movement in or out. The arrival of people into a cluster could dilute the impact of TB31F. Equally, if an individual who has received TB31F spends part of the year outside of the study cluster then the intervention-derived benefit will not be measurable within that cluster. Therefore, when powering the CRCT, it seems prudent to anticipate that only a proportion of TB31F’s impact will be measurable in each cluster. What value one should use for this proportion is beyond the scope of this work. However, it will be an important area of investigation in the future, as this will affect the evaluation of any transmission-blocking intervention. Here we will assume that 70% of the TB31F-derived benefit, in terms of malaria prevalence reduction, is measurable. Therefore, we power the study expecting a reduction in prevalence from 0.14 to 0.07.

An important factor in powering a CRCT is the between-cluster heterogeneity, here expressed in terms of the coefficient of variation, k (56). We consider an unmatched cluster-randomised trial and use standard methods to calculate sample sizes required to detect a significant difference (80% power, for a 5% significance level) in malaria prevalence between the control and intervention arms (ref Hayes & Moulton). Supplementary Figure 10B shows how the number of clusters per arm varies with k and the sample-size in each cluster. The vertical grey line indicates an (arbitrary) sample size of 100 in each cluster: we use this line to illustrate the number of clusters per arm required for 80% power, and how this varies with the value of k (the coloured lines). The dashed curves in this plot show the number of clusters per arm required for 80% power, assuming that the full benefit of TB31F could be measured in each cluster. This would enable a much smaller (and cheaper) CRCT to be carried out. It is clear, then, that the proportion of benefit that is measurable in a CRCT is a key factor to consider for transmission-blocking interventions. Note that here we have not considered certain logistical details that could be important for CRCTs, such as buffer zones between clusters (56). These would have to be considered carefully in practice.

Supplementary References

References 1-42 are numbered as per the main text.

43. Sheiner LB, Beal SL. Evaluation of methods for estimating population pharmacokinetic parameters II. Biexponential model and experimental pharmacokinetic data. J Pharmacokinet Biopharm. 1981 Oct 1;9(5):635–51.
44. Mould D, Upton R. Basic Concepts in Population Modeling, Simulation, and Model-Based Drug Development. CPT Pharmacometrics Syst Pharmacol. 2012 Sep;1(9):6.
45. Zhang L, Beal SL, Sheiner LB. Simultaneous vs. Sequential Analysis for Population PK/PD Data I: Best-Case Performance. J Pharmacokinet Pharmacodyn. 2003 Dec;30(6):387–404.
46. Dosne A-G, Bergstrand M, Harling K, Karlsson MO. Improving the estimation of parameter uncertainty distributions in nonlinear mixed effects models using sampling importance resampling. J Pharmacokinet Pharmacodyn. 2016 Dec 11;43(6):583–96.
47. World Health Organisation. WHO child growth standards: length/height-for-age, weight-for-age, weight-for-length, weight-for-height and body mass index-for-age: methods and development. 2006.
48. Kuczmarski RJ, Ogden CL, Guo SS, Grummer-Strawn LM, Flegal KM, Mei Z, et al. 2000 CDC Growth Charts for the United States: methods and development. Vital Health Stat 11. 2002 May;(246):1–190.
49. World Health Organisation. World Health Organisation Malaria Threats Map.
50. Sherrard-Smith E, Winskill P, Hamlet A, Ngufor C, N'Guessan R, Guelbeogo MW, et al. Optimising the deployment of vector control tools against malaria: a data-informed modelling study. Lancet Planet Heal. 2022 Feb;6(2):e100–9.
51. Bompard A, Da DF, Yerbanga RS, Biswas S, Kapulu M, Bousema T, et al. Evaluation of two lead malaria transmission blocking vaccine candidate antibodies in natural parasite-vector combinations. Sci Rep. 2017 Jul;7(1):6766.
52. Bompard A, Da DF, Yerbanga SR, Morlais I, Awono-Ambéné PH, Dabiré RK, et al. High Plasmodium infection intensity in naturally infected malaria vectors in Africa. Int J Parasitol. 2020;50(12):985–96.
53. Li H, Yu J, Liu C, Liu J, Subramaniam S, Zhao H, et al. Time dependent pharmacokinetics of pembrolizumab in patients with solid tumor and its correlation with best overall response. J Pharmacokinet Pharmacodyn. 2017 Oct 1;44(5):403–14.
54. Liu C, Yu J, Li H, Liu J, Xu Y, Song P, et al. Association of Time-Varying Clearance of Nivolumab With Disease Dynamics and Its Implications on Exposure Response Analysis. Clin Pharmacol Ther. 2017 May;101(5):657–66.
55. Baverel PG, Dubois VFS, Jin CY, Zheng Y, Song X, Jin X, et al. Population Pharmacokinetics of Durvalumab in Cancer Patients and Association With Longitudinal Biomarkers of Disease Status. Clin Pharmacol Ther. 2018 Apr 2;103(4):631–42.
56. Hayes RJ, Moulton LH. Cluster randomised trials. Chapman and Hall/CRC; 2017.

Supplementary Table 1. Final pharmacokinetic/pharmacodynamic model parameters

Model	Parameter	Estimate	95%CI SIR
Pharmacokinetic	Central volume (L)	2.57	2.19-3.00
	Peripheral volume 1 (L)	0.94	0.61-1.27
	Peripheral volume 2 (L)	1.47	1.17-1.78
	Clearance (L/h)	0.0051	0.0045-0.0058
	Inter-compartmental clearance 1 (L/h)	0.17	0.07-0.34
	Inter-compartmental clearance 2 (L/h)	0.0078	0.0034-0.0164
	Subcutaneous bioavailability (fraction)	0.54	0.45-0.67
	Absorption rate constant (h^{-1})	0.013	0.010-0.015
	IIV clearance (%CV)	30.0	22.9-41.2
	IIV central volume (%CV)	35.2	27.0-49.0
	Correlation clearance-central volume (%)	79	
	Proportional residual error (%)	18.5	16.9-20.0
Pharmacodynamic	Baseline oocyst count (n)	22.2	14.0-36.4
	Overdispersion parameter	0.445	0.416-0.475
	IC50 (mg/L)	2.18	2.12-2.24
	Hill factor	3.05	2.94-3.15
	IEV baseline count (%CV)	92.5	57.7-202

CI, confidence interval; CV, coefficient of variation; IIV, inter-individual variability; IC50, concentration at which 50% transmission-reducing activity is reached; IEV, inter-experiment variability; SIR, sampling importance resampling according to Dosne et al. (46). All pharmacokinetic parameters are allometrically scaled to a total body weight of 70 kg.

Supplementary Table 2: Cases averted per dose of TB31F administered. Here we report the clinical cases of malaria averted per dose of TB31F administered, for all modelling scenarios considered (as displayed in Figures 3 and 4). The impact of TB31F will depend on the age group targeted, as well as the other active public health interventions against malaria. In all scenarios, coverage of TB31F was 80% of the population targeted.

**High transmission site**		
Seasonal Malaria Chemoprevention ***not*** delivered to children aged 3-59 months		
	Age group targeted with TB31F	Cases averted per dose of TB31F administered
	School-aged children (5-15yrs)	0.89
	All children excluding infants (0.5-15yrs)	0.78
	Whole population excluding infants (>0.5 yrs)	0.66
Seasonal Malaria Chemoprevention delivered to children aged 3-59 months		
	Age group targeted with TB31F	Cases averted per dose of TB31F administered
	School-aged children (5-15yrs)	0.77
	All children excluding infants (0.5-15yrs)	0.55
	Whole population excluding infants (>0.5 yrs)	0.49
**Low transmission site**		
Seasonal Malaria Chemoprevention ***not*** delivered to children aged 3-59 months		
	Age group targeted with TB31F	Cases averted per dose of TB31F administered
	School-aged children (5-15yrs)	0.39
	All children excluding infants (0.5-15yrs)	0.30
	Whole population excluding infants (>0.5 yrs)	0.27
Seasonal Malaria Chemoprevention delivered to children aged 3-59 months		
	Age group targeted with TB31F	Cases averted per dose of TB31F administered
	School-aged children (5-15yrs)	0.33
	All children excluding infants (0.5-15yrs)	0.22
	Whole population excluding infants (>0.5 yrs)	0.21

Pharmacokinetic/pharmacodynamic goodness-of-fit plots

Supplementary Figure 1. (**a**) Population and (**b**) individual model-predicted versus observed antibody concentrations. The solid black lines represent the line of unity.

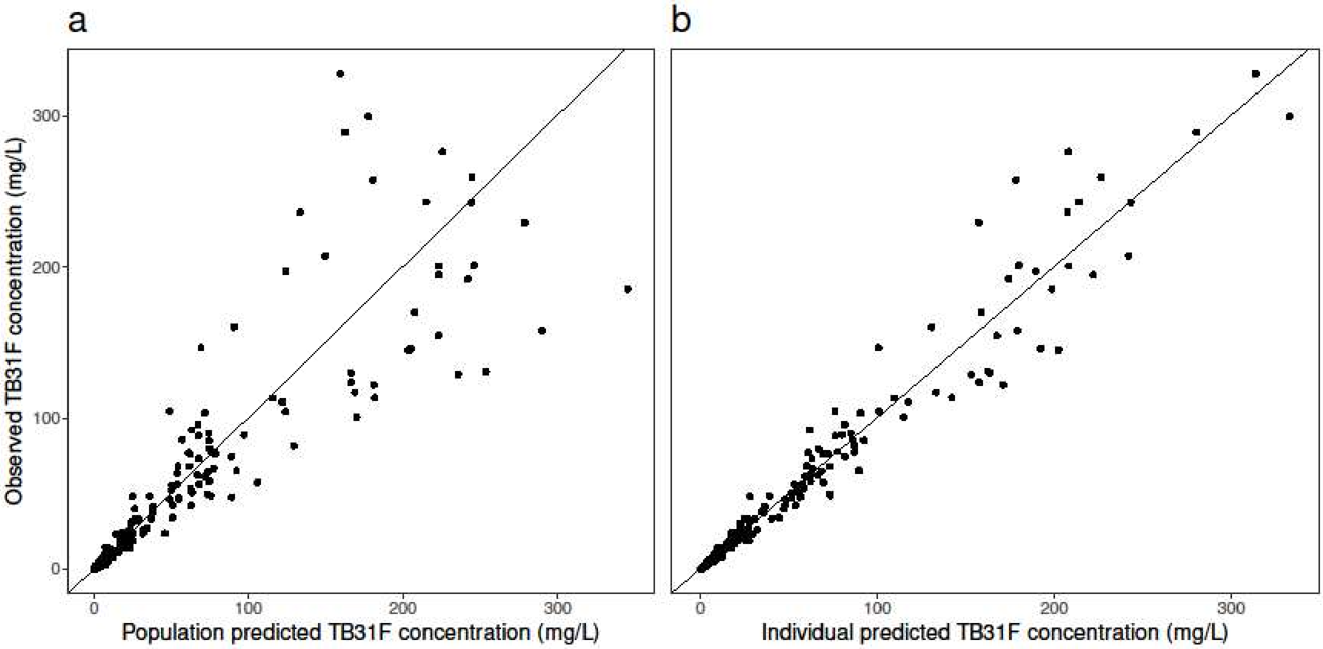

Supplementary Figure 2. (**a**) Population model-predicted antibody concentration and (**b**) time after start of treatment versus conditional weighted residuals with interaction (CWRESI). The data points (dots) are evenly scattered around the zero-line (solid black lines) as shown by the smooth function (solid blue lines).

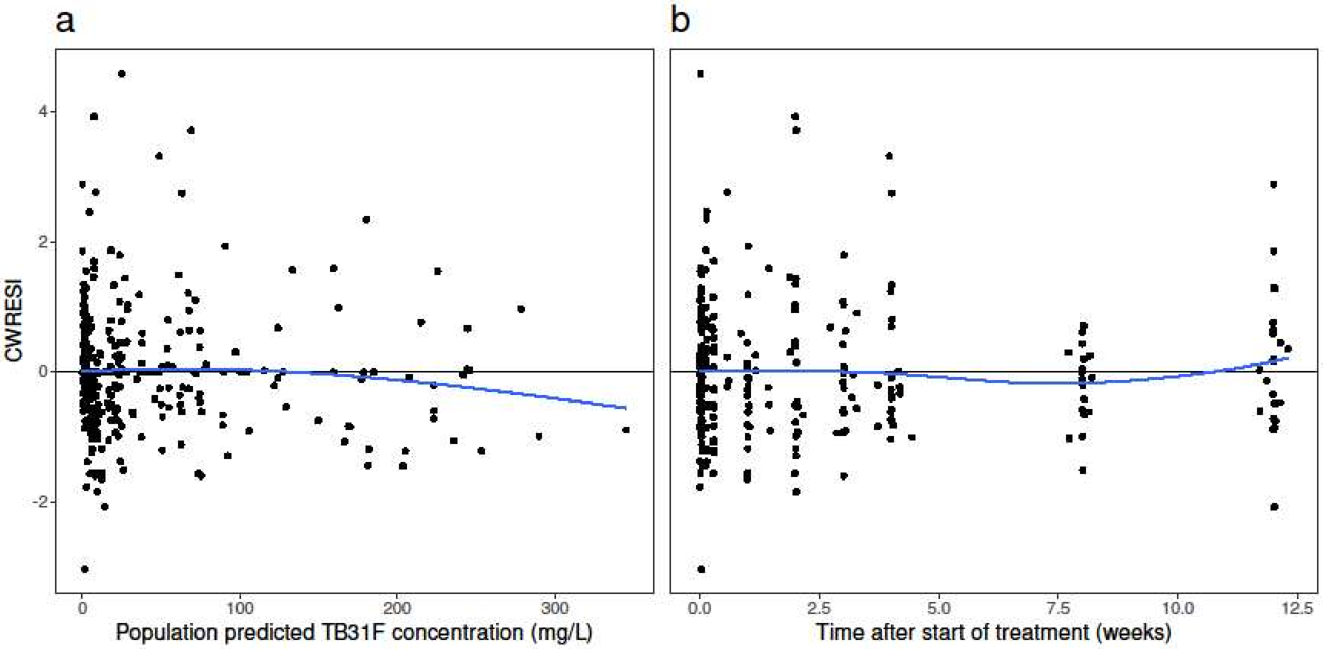

Supplementary Figure 3. Observed and simulated TB31F concentrations over time per dosing group. The solid lines represent the 50th percentiles of the observed TB31F concentrations depicted by the open circles. The shaded areas represent the simulation-based 95% confidence intervals of the 50th percentiles based on n=1000 simulations.

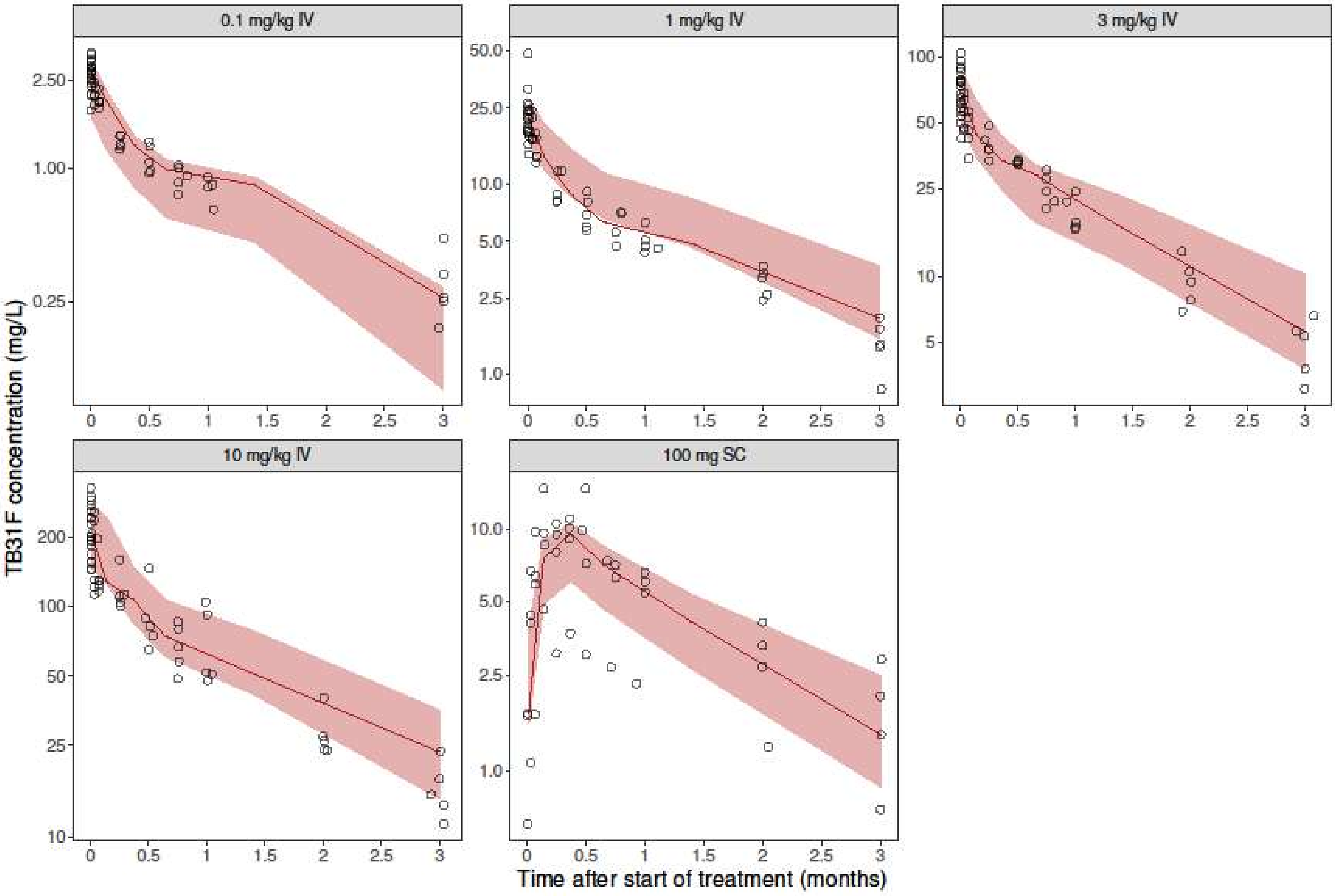

Supplementary Figure 4. Prediction-corrected visual predictive check of the pharmacokinetic model based on n=1000 simulations. Observed and simulated TB31F concentrations are normalized to make them comparable between dosing groups (Bergstrand et al.). The solid line represents the 50th percentile and the dashed lines represent the 2.5th and 97.5th percentiles of the observed TB31F concentrations depicted by the open circles. The shaded areas represent the simulation-based 95% confidence intervals of the 2.5th, 50th, and 97.5th percentiles.

Bergstrand M, Hooker AC, Wallin JE, Karlsson MO. Prediction-corrected visual predictive checks for diagnosing nonlinear mixed-effects models. AAPS J. 2011 Jun;13(2):143-51.

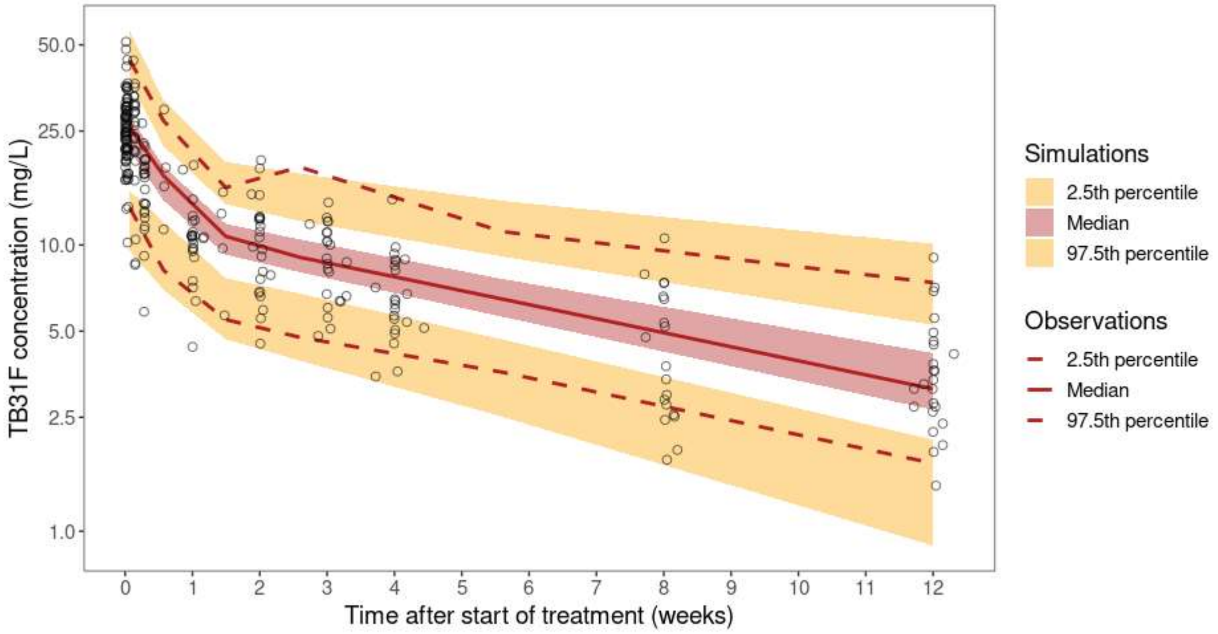

Supplementary Figure 5. Observed and simulated antibody concentration versus the probability of an oocyst count of 0. The solid blue line represents the observed probability. The red band represents the 95% prediction interval of the simulated probability based on n=1000 simulations.

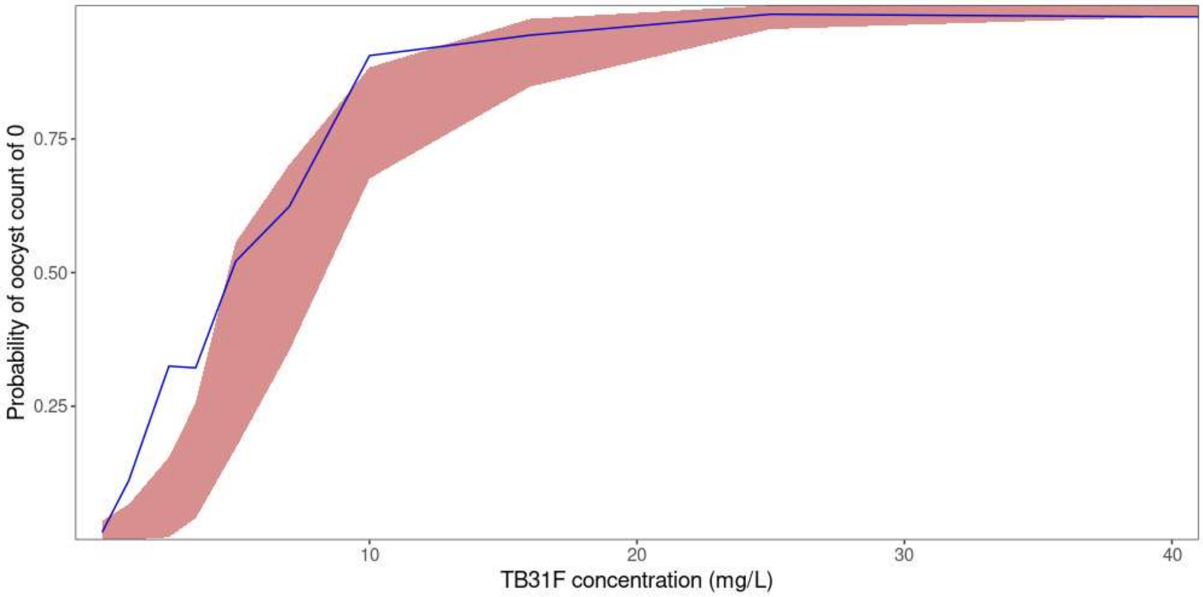

Supplementary Figure 6. The changing infectious reservoir of malaria at the community level, as interventions are introduced (including the introduction of TB31F to all age groups). Here we show how the introduction of public health interventions against malaria can reduce the infectious reservoir at the community level. We show contributions to the infectious reservoir made by three age groups: children under five years of age, school-aged children, and adults. These contributions are influenced by the average per-person infectivity (y-axes), as well as relative sizes of the three subpopulations (x-axes). Panels A-C: modelling results from a low transmission setting, based upon the Upper River region of the Gambia. Panels D-F: modelling results from a high transmission setting, based upon the Sahel region of Burkina Faso. For both settings, we first assessed the infectious reservoir prior to the introduction of seasonal malaria chemoprevention (SMC) or TB31F, i.e. insecticide-treated nets (ITNs) are the only intervention in use (panels A and D). We then assessed the impact of delivering SMC to 80% of children between 3 and 59 months of age (panels B and E). Thirdly, we measure the impact of delivering TB13F to 80% of all age groups (panels C and F, excluding children under 6 months of age). The percentages above each bar indicates the contribution that each age group makes to the infectious reservoir. The percentage reductions (grey text) displayed in the top-right corner of the middle and right panels indicate the overall reduction in the infectious reservoir relative to the ITN-only scenario in each setting. For all results, a generic demography for sub-Saharan Africa was used, as per Griffin et al. (21). Note that the results presented here are not adjusted for age-dependent biting of mosquitoes.

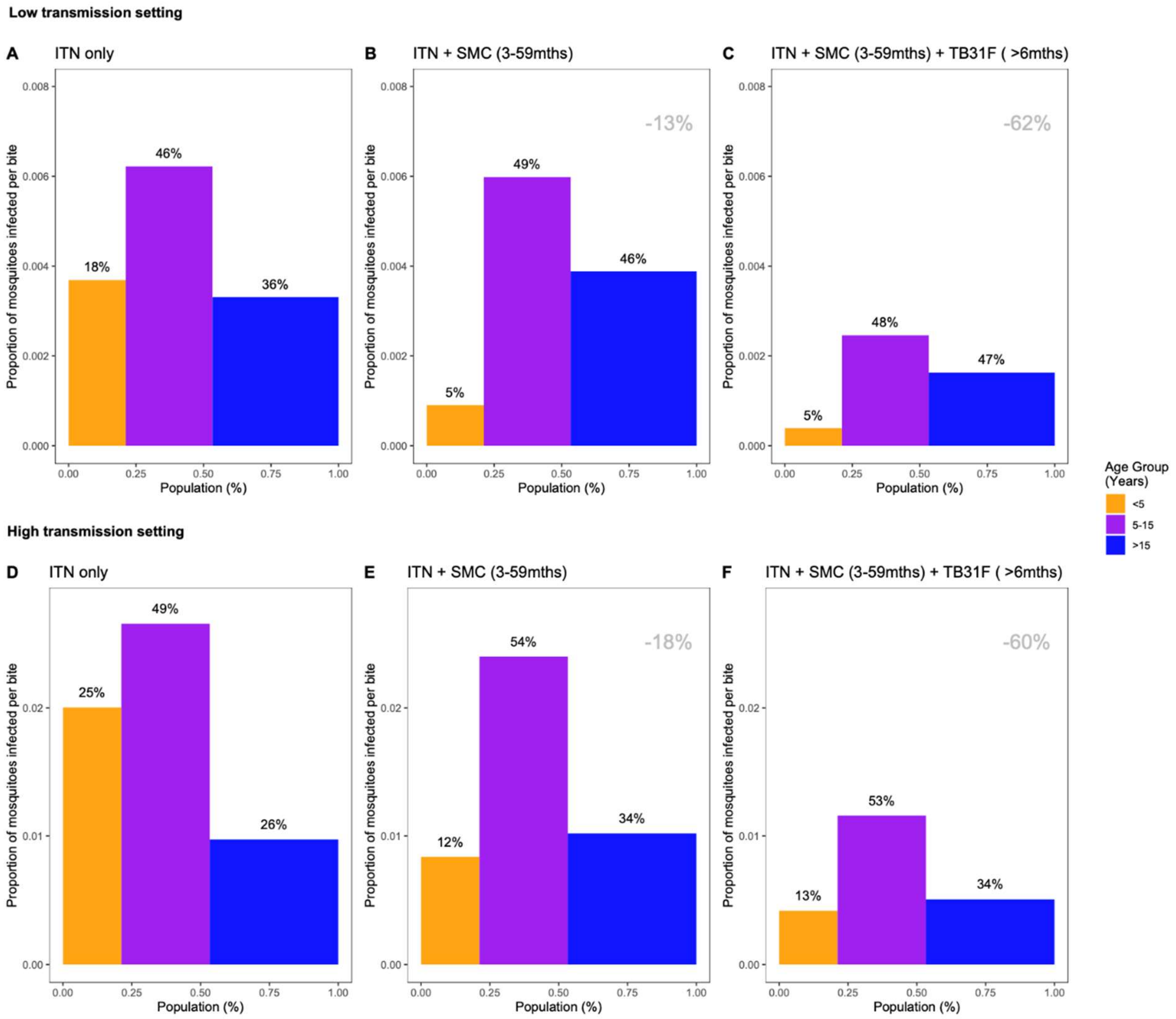

Supplementary Figure 7. How changing the relationship between transmission-reducing activity and transmission-blocking activity affects the epidemiological impact of TB31F. In order to model TB31F as a public health intervention, we had to translate the measured pharmacodynamic properties of TB31F, calculated in terms of a transmission-reducing activity (TRA, Figure 2A), into a predicted efficacy for blocking human-to-vector transmission events in the field. This translation utilised data on oocyst counts in naturally infected, wild mosquitoes. This data was collected in a high transmission setting in Burkina Faso in 2014 (52), and was used to generate a relationship between TRA and transmission-blocking activity (TBA) in recent modelling work (23), which is shown by the black curve in panel A. This is the TRA-TBA relationship used throughout this work. The distribution of oocyst counts, which determines this relationship, is likely to vary across different malaria-endemic settings. Therefore, we varied the mean of this distribution (which is a zero-truncated negative binomial distribution), keeping the dispersion parameter fixed. The black curve is generated from a zero-truncated oocyst distribution, characterised by parameters m=0.000157 and k=0.00000495 (mean oocyst count=9.1). We double the mean of the distribution by setting m=0.000398, resulting in a TRA-TBA relationship shown by the orange curve in panel A. We quarter the mean, by setting m=0.0000158, resulting in a TRA-TBA relationship shown by the green curve in panel A. Panels B and C show the public health impact of the modelled administrations of TB31F, as described in Figures 3 and 4, in the high- and low-transmission settings, respectively. Decreasing the mean oocyst counts increases the impact of the public health interventions, and *vice versa* (changes indicated by the error bars). Therefore, the public health projections are robust to changes in the TRA-TBA relationship.

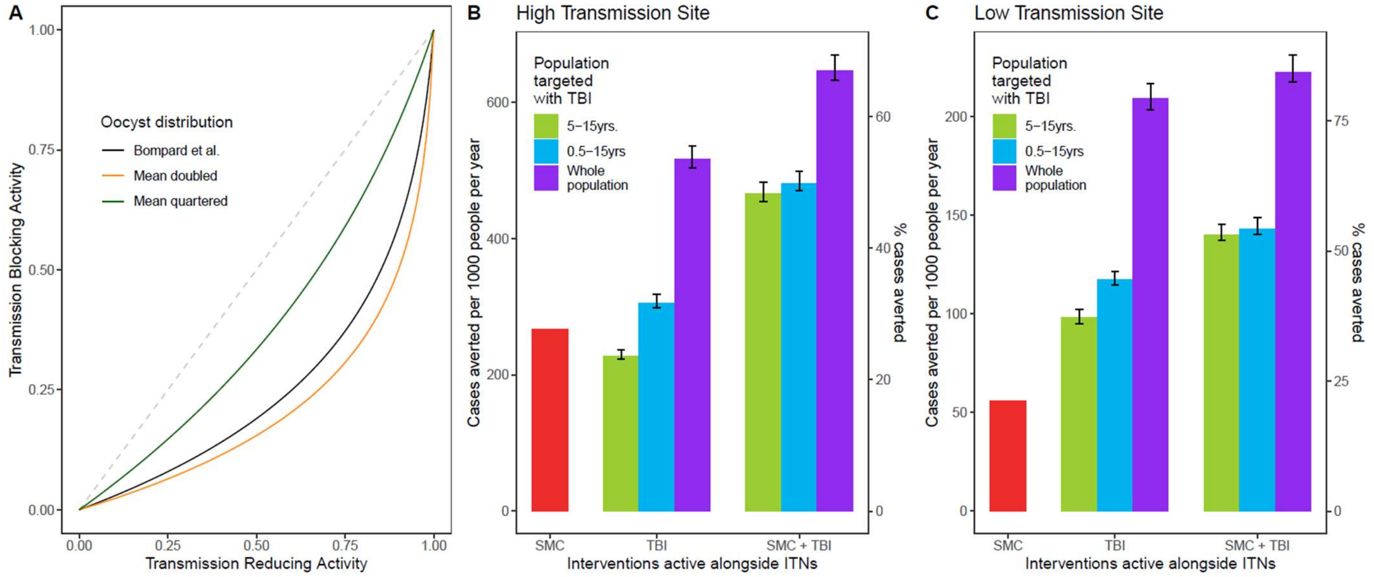

Supplementary Figure 8. Impact of adjusting the clearance parameter in the pharmacokinetic model to account for a potentially faster clearance in the target population. In this sensitivity analysis, we explored the impact of a 1.33-fold increase in the population-average clearance parameter (that is, an increase from 0.0051 L/h to 0.0068 L/h) on the public health impact estimates of TB31F. Panel A: an increased clearance reduces the amount of time that that a transmission-reducing activity (TRA) of >80% can be maintained. These TRA projections were generated from PK modelling of two simulated cohorts, each of 4000 school-aged children. The age-distribution of these cohorts was consistent with that used in the transmission model (an exponential distribution with a mean of 21 years). Panels B and C: public health impact of increased clearance, in the high- and low-transmission settings, respectively. The modelling scenarios are as described in Figures 3 and 4. The original results, presented in Figures 3 and 4, are indicated by the grey, dashed bars. This indicates that the impact of TB31F would be slightly impacted by increased clearance in the cohort chosen for administration. One reason the impact is quite small is that the two settings chosen here are highly seasonal: this means that most clinical malaria occurs within the first 3 months after administration. During this time period, the impact of increased clearance upon the TRA of the modelled cohort is not so apparent: we expect a larger impact to appear, if TB31F was administered in a setting that was less seasonal.

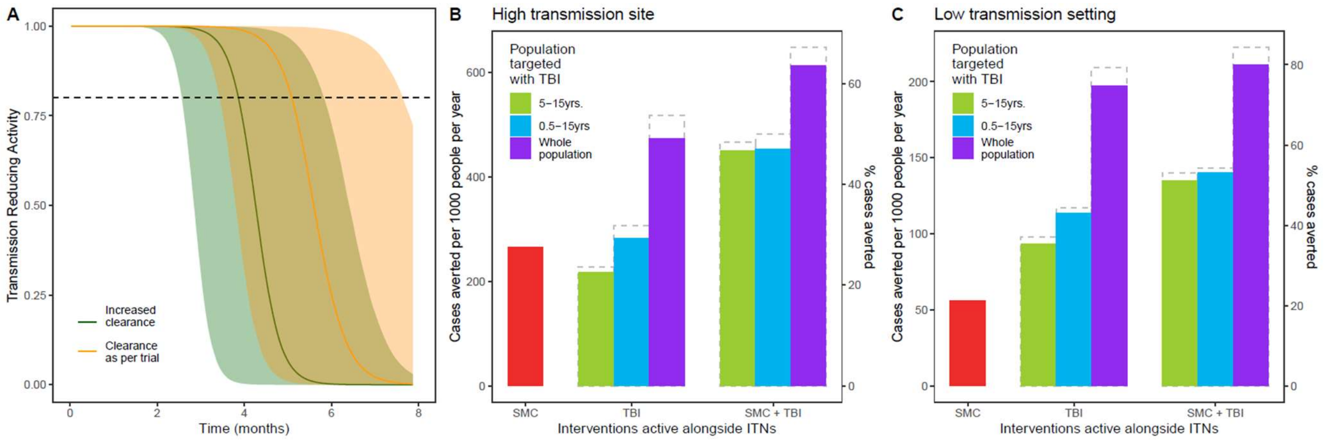

Supplementary Figure 9: A sensitivity analysis for the relationship between TB31F concentration and transmission reducing activity (TRA). Panel A: here we show that the relationship between TRA and the antibody concentration is relatively robust to large changes in the IC50 parameter (see Supplementary Methods, Supplementary Table 1). The TRA for the measured IC50 is shown in dark green, we also show the TRA when the IC50 is increased by 50% (orange) and 100% (blue). The inset of the panel shows the same plot over a narrower range of antibody concentration: here we also show the uncertainty in the fitted parameter values for IC50 and the Hill factor (Supplementary Table 1), with the dark green shaded area indicating the 95% confidence intervals for the TRA. Panel B: How changing the relationship between TRA and antibody concentration alters the duration of TB31F's activity. Using the fitted pharmacokinetic model, we simulated the antibody concentrations over time for 4000 school-aged children. We then used the three relationships shown in panel A (same colours used in this panel) to find the duration of TRA in each case. The solid lines show the median duration of TRA for each model, with the dashed lines indicating the 95% intervals. The horizontal dashed line indicates a TRA of 80%. Comparing this panel with the results shown in Supplementary Figure 8, we can see that increasing IC50 has a smaller impact than increasing the clearance parameter in the pharmacokinetic model. Therefore, we can safely conclude that the public health impact of TB31F is robust to changes in this facet of our modelling approach.

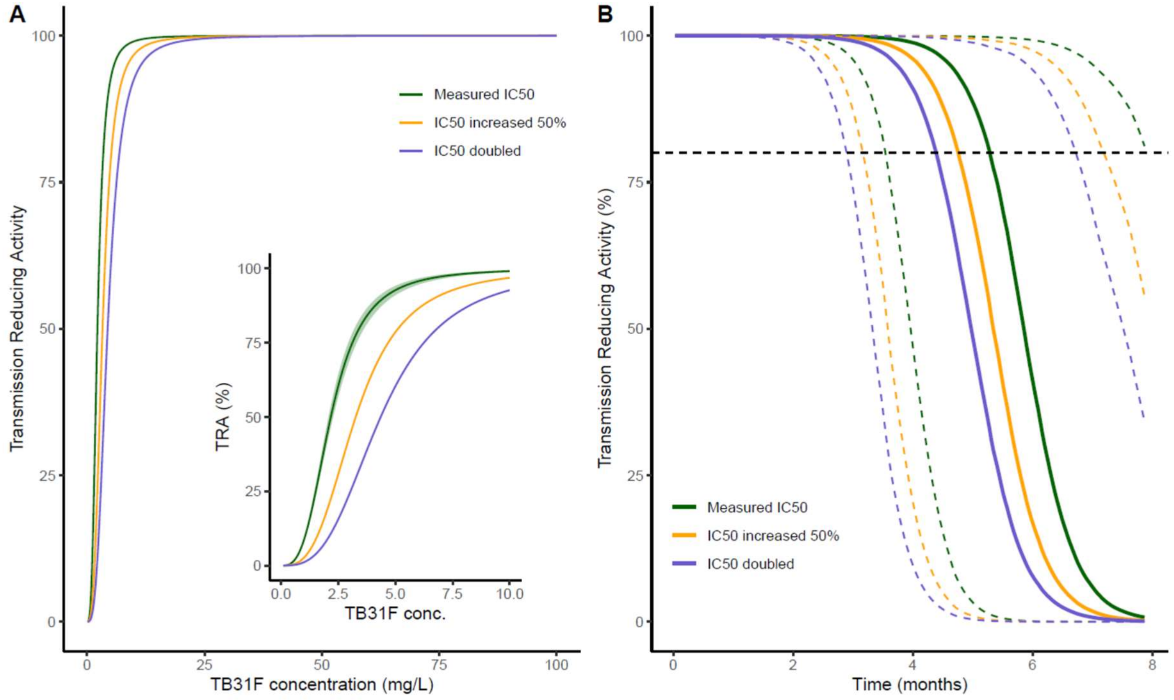

Supplementary Figure 10: Using mathematical modelling to design a hypothetical cluster randomised trial for TB31F. Panel A: modelled malaria prevalence over time, with different active interventions. This setting is based on the Upper River region of the Gambia, as used in Figures 5. We here use the model-predicted impact of TB31F, when used alongside insecticide-treated nets and seasonal malaria chemoprevention in children aged 3-59 months, to calculate the sample size required to measure a reduction in prevalence (with 80% power at the 5% significance level) in a cluster-randomised trial. In this scenario, clusters are randomised to receive either TB31F or a placebo, which is administered to all age groups (excluding children under 6 months of age) at 80% prevalence. This CRCT would run over two malaria transmission seasons, and malaria prevalence would be measured at the end of the second season (dashed, vertical grey line). The transmission model predicts a reduction in malaria prevalence from 0.14 (black curve) to 0.04 (red curve) at this time point. We assume that 70% of this benefit would be measurable in a CRCT. Panel B: sample size requirements for the CRCT for varying cluster size. Here we show how the between-cluster heterogeneity (here measured in terms of the coefficient of variation, *k*) and the cluster size influences the number of clusters per arm required to power the trial. In the case where the malaria prevalence is measured in 100 individuals in each cluster, the coloured numbers near the y-axis indicate the required number of clusters per arm (the colours correspond to the value of *k*), rounded to the nearest integer. The dashed lines show that a smaller CRCT could be carried out, if the full benefit of TB31F were measurable in the trial. While measuring the full benefit may not be realistic in practice, these results indicate that the proportion of benefit that is measurable in the trial is an important factor to consider.

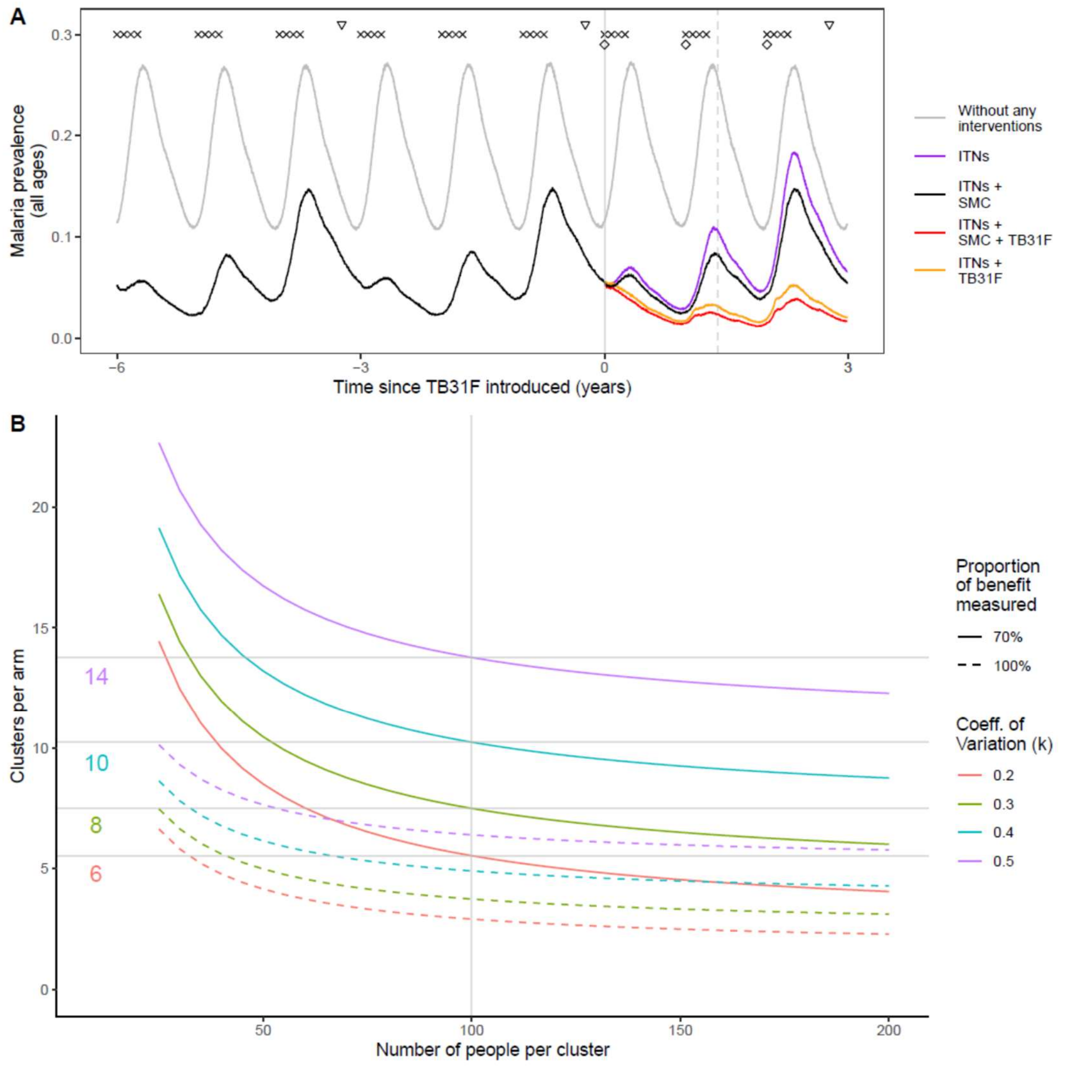

NONMEM control stream for the final pharmacokinetic model

```
$PROB TB31F
$INPUT GROUP ID TIME AMT RATE DV EVID CMT BLQ SEX HT AGE WT
$DATA TB31F_NM.csv IGNORE=@
$SUBROUTINE ADVAN13 TOL=6

$MODEL NCOMPARTMENTS=4
COMP=(CENTRAL)
COMP=(PERI)
COMP=(DEPOT)
COMP=(PERI2)

$PK
WTCL = (WT/70)**0.75
WTV  = (WT/70)**1

TVCL=THETA(1)                          ; TYPICAL CLEARANCE
CL=TVCL*EXP(ETA(1))*WTCL               ; INDIVIDUAL CLEARANCE
F1=1
TVV1=THETA(2)                          ; TYPICAL V1
V1=TVV1*EXP(ETA(2))*WTV                ; V1
TVV2=THETA(3)
V2=TVV2*WTV
TVQ=THETA(4)
Q=TVQ*WTCL
TVV3=THETA(7)
V3=TVV3*WTV
TVQ2=THETA(8)
Q2=THETA(8)*WTCL

S1=V1
S2=V2
S3=V3

K10=CL/V1
K12=Q/V1
K21=Q/V2
K14=Q2/V1
K41=Q2/V3

; SC parameters
F3 = THETA(5)
KA = THETA(6)

$DES
DADT(1) = K21*A(2) - K12*A(1) - K10*A(1) + KA*A(3) - K14*A(1) + K41*A(4)
DADT(2) = K12*A(1) - K21*A(2)
DADT(3) = -KA*A(3)
DADT(4) = K14*A(1) - K41*A(4)

$ERROR
IPRED=F
Y=IPRED+IPRED*ERR(1)
IRES = DV - IPRED
IWRES = IRES/IPRED

$THETA (0, 0.0051)          ; CL L/H
$THETA (0, 2.57)            ; V1 L
$THETA (0, 0.944)           ; V2
$THETA (0, 0.172)           ; Q
$THETA (0, 0.541, 1)        ; F
$THETA (0, 0.0128)          ; KA
$THETA (0, 1.47)            ; V3
$THETA (0, 0.0078)          ; Q2

$OMEGA BLOCK(2)
0.0863                      ; IIV CL
0.0797 0.117                ; IIV V1

$SIGMA 0.0337

$ESTIMATION METHOD=1 INTER MAXEVAL=9999 NSIG=3 SIGL=9 PRINT=3
$COVARIANCE PRINT=E MATRIX=S

$TABLE ID GROUP TIME CMT AMT RATE DV EVID WT SEX HT AGE BLQ CL V1 V2 Q IPRED PRED CWRES CWRESI IWRES ETA1 ETA2 NOPRINT ONEHEADER
NOAPPEND FILE=sdtab1
```

NONMEM control stream for the final pharmacodynamic model

```
$PROB TB31F
$INPUT ID GROUP SUB MOSN OOCT DV MOSNT TIME CONC EVID CMT BLQ AMT RATE SEX HT AGE WT IIN LST
$DATA TB31F_NM_PD.csv IGNORE=@

$ABBREV PROTECT

$PRED
LCONC = -2
IF(CONC.NE.0) LCONC = LOG10(CONC)
EMAX = 1
EC50 = THETA(1)
HILL = THETA(2)
TVBASE = THETA(3)
BASE   = TVBASE * EXP(ETA(1))
DE     = EMAX*(CONC**HILL)/((CONC**HILL)+(EC50**HILL))
LAMB = BASE * ( 1- DE )
OVDP = THETA(4)          ;overdispersion factor

IF(DV.LE.1) THEN
LFAC=0
ELSE
LFAC = DV*LOG(DV)-DV +LOG(DV*(1+4*DV*(1+2*DV)))/6 +LOG(3.1415)/2
ENDIF

  ;gamma functions of the negative binomial model expression
 LGAM1=LOG(SQRT(2*3.1415))+((DV+1/OVDP)-0.5)*LOG((DV+1/OVDP))-(DV+1/OVDP)+LOG(1+1/(12*(DV+1/OVDP)))
 LGAM2=LOG(SQRT(2*3.1415))+((1/OVDP)-0.5)*LOG((1/OVDP))-(1/OVDP)+LOG(1+1/(12*(1/OVDP)))

 LTRM1=(LOG(1/(1+OVDP*LAMB)))*(1/OVDP)
 LTRM2=(LOG(LAMB/(LAMB+1/OVDP)))*(DV)

  ;Logarithm of the Negative Binomial distribution
 LNB = LGAM1-LFAC-LGAM2+LTRM1+LTRM2  ;Ln(negative binomial)
  ;-2 Log Likelihood
 Y=-2*LNB

$THETA (0, 2.18)                ; 1 EC50
$THETA (0, 3.05)                ; 2 HILL
$THETA (0, 22.2)                ; 3 BASELINE OOCYSTS
$THETA (0, 0.445)               ; 4 OVDP

$OMEGA 0.618                    ; 1 inter-assay variability BASELINE OOCYSTS

$ESTIMATION METHOD=COND LAPLACE -2LL MAXEVAL=9999 NSIG=3 SIGL=9 PRINT=3
$COVARIANCE PRINT=E MATRIX=S

$TABLE ID GROUP SUB IIN MOSN TIME CONC DV WT SEX HT AGE LAMB PRED LST ETA1 BASE LCONC NOPRINT ONEHEADER NOAPPEND FILE=sdtab1
```
